# The Prophage and Plasmid Mobilome as a Likely Driver of Mycobacterium abscessus Diversity

**DOI:** 10.1128/mBio.03441-20

**Published:** 2021-03-30

**Authors:** Rebekah M. Dedrick, Haley G. Aull, Deborah Jacobs-Sera, Rebecca A. Garlena, Daniel A. Russell, Bailey E. Smith, Vaishnavi Mahalingam, Lawrence Abad, Christian H. Gauthier, Graham F. Hatfull

**Affiliations:** a Department of Biological Sciences, University of Pittsburgh, Pittsburgh, Pennsylvania, USA; University of Massachusetts Amherst

**Keywords:** *Mycobacterium abscessus*, prophages, bacteriophages, plasmids

## Abstract

Mycobacterium abscessus is an emerging pathogen that is often refractory to antibiotic control. Treatment is further complicated by considerable variation among clinical isolates in both their genetic constitution and their clinical manifestations. Here, we show that the prophage and plasmid mobilome is a likely contributor to this variation. Prophages and plasmids are common, abundant, and highly diverse, and code for large repertoires of genes influencing virulence, antibiotic susceptibility, and defense against viral infection. At least 85% of the strains we describe carry one or more prophages, representing at least 17 distinct and diverse sequence “clusters,” integrated at 18 different *attB* locations. The prophages code for 19 distinct configurations of polymorphic toxin and toxin-immunity systems, each with WXG-100 motifs for export through type VII secretion systems. These are located adjacent to attachment junctions, are lysogenically expressed, and are implicated in promoting growth in infected host cells. Although the plethora of prophages and plasmids confounds the understanding of M. abscessus pathogenicity, they also provide an abundance of tools for M. abscessus engineering.

## Abstract (precis)

Mycobacterium abscessus is an important emerging pathogen that is challenging to treat with current antibiotic regimens. There is substantial genomic variation in M. abscessus clinical isolates, but little is known about how this influences pathogenicity and *in vivo* growth.

## INTRODUCTION

Bacteriophages are characteristically specific for their bacterial hosts, with preferences that rarely traverse genus boundaries and are sometimes constrained to only a subset of isolates within a bacterial species (1). Phage specificity is determined by numerous factors, including receptor accessibility, restriction-modification, CRISPR-Cas, and abortive-infection systems, many of which can be expressed from prophages or plasmids (2–6). Because prophages and plasmids are highly mobile, these are key contributors to variations in phage infection among otherwise closely related bacterial strains. For using phages therapeutically to control bacterial infections, this specificity is a double-edged sword; it facilitates targeting of particular pathogens without gross microbiome disturbance, but constrains the range of bacterial isolates sensitive to any particular phage (7).

A large collection of mycobacteriophages have been isolated on Mycobacterium smegmatis and genomically characterized (8). They are genetically diverse and are currently grouped into 29 clusters (A to Z, AA to AC) according to overall sequence relatedness, many of which can be further divided into subclusters (e.g., A1, A2, A3, etc.). Additionally, there are 10 “singletons,” each with no close relative (9–11). More than 50% of these groups contain temperate phages, many of which code for prophage-mediated phage defense systems that interfere with heterotypic (unrelated) phages, often with exquisite specificity (3, 12, 13). A small subset of clusters/subclusters (A2, A3, G, K) also infect Mycobacterium tuberculosis, suggesting these have broader host ranges than the many that do not (14). Very few of the phages infect Mycobacterium abscessus, but a cocktail of three phages within this subset were used for therapy of a disseminated drug-resistant infection in a cystic fibrosis patient with a bilateral lung transplant (15); two of the phages were engineered to convert from temperate to being obligatorily lytic (15) using a recombineering strategy (16).

Clinical isolates of M. abscessus differ greatly in their phage infection profiles, presenting challenges in broadening phage therapeutic applications (17). However, the phage infection profiles do not correlate with whole-genome phylogenies, and mobile elements, including prophages and plasmids, are likely major contributors (18, 19). To understand the potential roles of the mycobacterial mobilome in these properties, we have characterized the prophages and plasmids of a recently genomically defined set of 82 recent M. abscessus clinical isolates with well-defined phage infection profiles (17).

## RESULTS

### Identification of M. abscessus prophages and plasmids.

**FIG 1:**
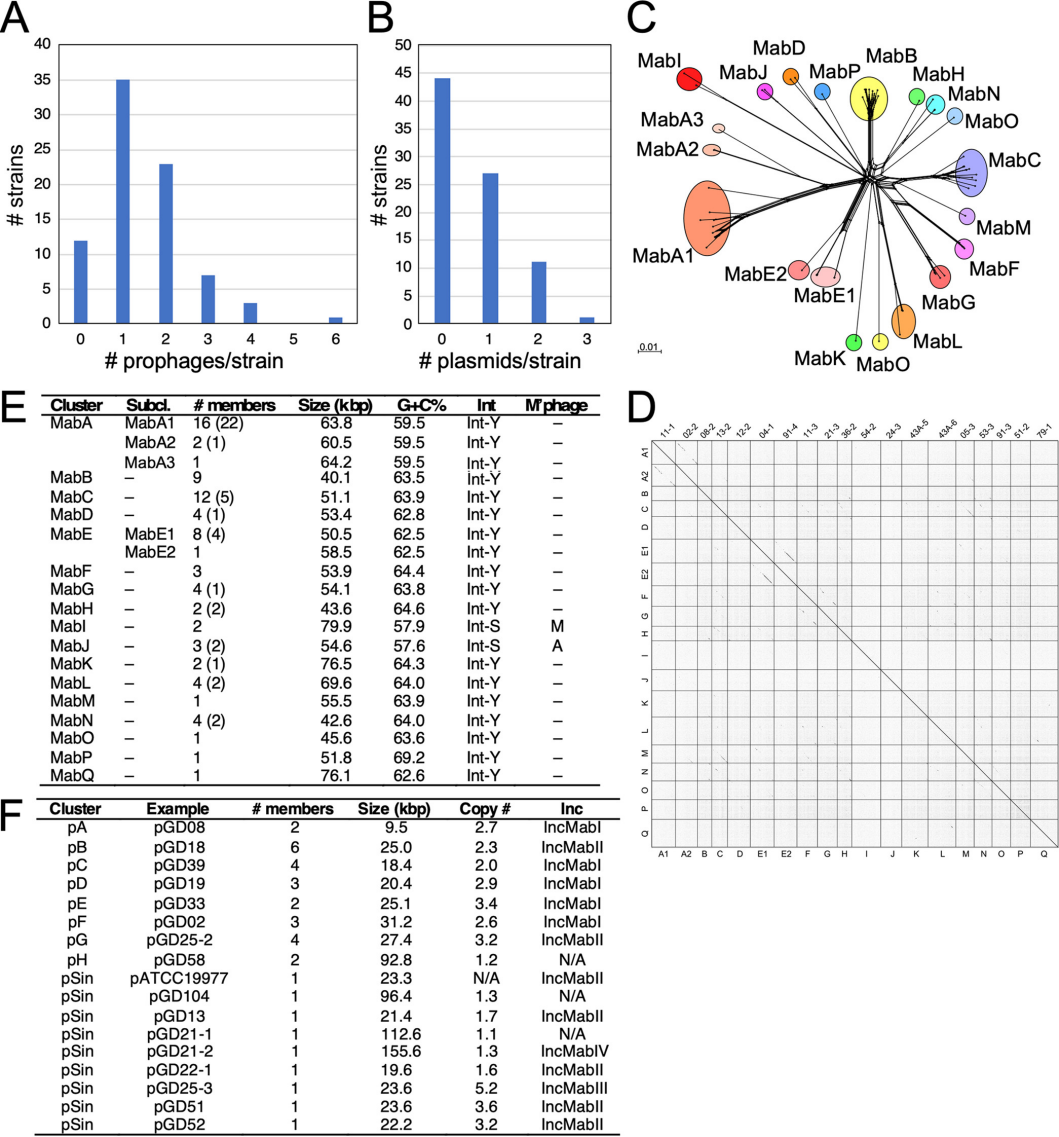
Diversity of M. abscessus prophages and plasmids. (A and B) Distributions of prophages (A) and plasmids (B) in 82 recent M. abscessus clinical isolates. (C) Phylogenetic network representation of M. abscessus prophages based on shared gene content, as described elsewhere (9, 63). Individual prophages are represented at the nodes, and colored circles indicate groups of phages forming clusters. Scale marker indicates substitutions/site. (D) Dotplot comparison of M. abscessus prophages, comparing one example of each cluster and subcluster, and indicated on both axes. Individual genes are noted at the top. (E) Characteristics of M. abscessus prophages showing the numbers of members in each cluster/subcluster group (the number of additional incomplete prophage sequences are shown in parentheses), average genome size in kbp, average G+C% content, presence of a tyrosine-family (Int-Y) or serine-family (Int-S) integrase, and distantly related mycobacteriophage (M’phage) clusters. (F) Characteristics of M. abscessus plasmids showing examples, the numbers of members in each cluster, average genome size in kbp, average copy number, and the predicted incompatibility (Inc) group.

Using a panel of 82 recent clinical isolates of M. abscessus, we identified prophages using PHASTER (20) and manual inspection to precisely map the prophage junctions with conserved common core sequences at both *attL* and *attR*. We identified a total of 122 prophages, 80 for which complete genome sequences could be extracted (Table 1). Each was given a prophiGDxx-# designation according to the parental strain with a numerical suffix to denote multiple prophages in a single strain (Table 1). There are several instances of identical prophages present in different strains, and 67 unique prophage sequences were identified (Table 1); 42 prophages are in multiple contigs, but sufficient sequence information is available to indicate their relationships to other prophages (17). We also extracted prophage sequences from M. abscessus subsp. *abscessus* ATCC 19977 (21) and M. abscessus subsp. *bolletii* BD^T^ (22); M. abscessus subsp. *massiliense* GO06 (23) is prophage-free (Table 1). The prophage in ATCC 19977 was previously reported to be 81 kbp (21), but is about 20 kbp shorter. Identical prophages are present in strains GD26, GD47, and GD40, and the *attL* and *attR* sites were confirmed by comparison to lytically growing phage relatives (17). Twelve (15%) of the strains are prophage-free, and the other 70 contain 1 to 6 prophages (Fig. 1A). The 75 complete prophages vary in size from 39,188 bp (prophiGD62-1) to 80,793 (prophiGD86-1), with an average size of ∼55.3 kbp. Identical prophages are present in some strains from different origins with phylogenetically distinct genomes, reflecting the high phage mobility within this group of bacteria. We similarly identified resident plasmids, all of which are extrachromosomal and circular with the exception of pGD21-2, which is linear (Fig. 1B; Table 2). Approximately one-half of the strains are plasmid-free, and the others have 1 to 3 different plasmids (Fig. 1B).

**TABLE 1:** Prophages resident in M. abscessus genomes

Prophage^*a*^	Cluster^*b*^	attB^*c*^	Coordinates^*d*^	Length (bp)^*e*^	ORFs	tRNA (tmRNA)	Accession no.^*f*^	M’phage^*g*^
prophiATCC19977-1	MabA1	attB-5	1754373–1816169	61,797	113	0	CU458896	–
prophiGD26-1	MabA1	attB-5	C2 475612–537408	61,797	113	0	CP063319	
prophiGD47-1	MabA1	attB-5	C3 292175–353971	61,797	113	0	JADWXG000000000	
prophiGD40-1	MabA1	attB-5	C3 292175–353971	61,797	113	0	JADWXE000000000	
prophiGD11-1	MabA1	attB-5	C5 181029–253263	62,235	115	0	MW584152	–
prophiGD15-1	MabA1	attB-5	C2 233283–162702	70,582	124	0	MW584180	–
prophiGD41-1	MabA1	attB-5	C2 292146–362727	70,582	124	0	CP065283	
prophiGD59-1	MabA1	attB-5	C2 292146–362727	70,582	124	0	CP065274	
prophiGD17-2	MabA1	attB-5	1784635–1847562	61,797	113	0	MW584161	–
prophiGD20-1	MabA1	attB-5	1737838–1806869	69,032	129	0	MW584158	–
prophiGD21-2	MabA1	attB-5	209421–147437r	61,985	117	0	MW584204	–
prophiGD22-1	MabA1	attB-5	1753803–1814377	60,575	118	0	MW584171	–
prophiGD27-1	MabA1	attB-5	C3 308161–367705	59,545	116	0	MW584196	–
prophiGD43A-1	MabA1	attB-18	1665669–1727971	62,303	118	0	MW584179	
prophiGD57-2	MabA1	attB-5	1753840–1813851	60,012	117	0	MW584164	–
prophiGD102-1	MabA1	attB-5	429593–366359	63,235	117	0	MW584211	–
prophiGD02-2	MabA2	attB-15	C2 220134–280685	60,552	115	0	MW584199	–
prophiGD90-1	MabA2	attB-15	C15 62013–1462r	60,552	115	0	MW584176	–
prophiGD91-2	MabA3	attB-5	1820105–1884312	64,208	117	0	MW584188	–
prophiGD08-2	MabB	attB-2	C14,108963–68889r	40,071	63	0	MW584184	–
prophiGD11-2	MabB	attB-2	C14,108999–69045r	39,951	63	0	MW584151	–
prophiGD16-1	MabB	attB-2	C9, 46031–86086	40,056	59	0	MW584149	–
prophiGD21-1	MabB	attB-2	C10 36362–77136	40,775	63	1	MW584205	–
prophiGD34-2	MabB	attB-2	C13 109092–69049	40,044	63	0	MW584210	–
prophiGD42-2	MabB	attB-2	C14 35327–75397	40,071	63	0	MW584200	–
prophiGD43A-2	MabB	attB-2	567429–607575	40,147	61	0	MW584198	–
prophiGD62-1	MabB	attB-2	C4 254446–293633	39,188	59	0	MW584194	–
prophiGD89-1	MabB	attB-2	C12 48033–88524	40,492	63	0	MW584193	–
prophiGD13-2	MabC	attB-13	C1 743264–794604	51,341	70	0	MW584212	–
prophiGD33-1	MabC	attB-12	C4 300386–351461	51,076	71	0	MW584203	–
prophiGD39-2	MabC	attB-13	C1 414064–361484r	52,581	71	0	MW584154	–
prophiGD43A-3	MabC	attB-13	4129439–4180188	50,750	71	0	MW584182	–
prophiGD44-1	MabC	attB-13	C1 275695–222635r	53,061	76	0	MW584156	–
prophiGD51-1	MabC	attB-13	C1 756876–808079	51,204	77	0	MW584163	–
prophiGD52-1	MabC	attB-13	C2 403351–453146	49,796	73	0	MW584208	–
prophiGD57-1	MabC	attB-13	4001908–4054497	52,581	73	0	MW584181	–
prophiGD91-1	MabC	attB-13	4267862–4317619	49,758	72	0	MW584192	–
prophiGD100A-2	MabC	attB-12	617106–667933	50,828	71	0	MW584150	–
prophiGD100B-2	MabC	attB-12	617106–667933	50,828	71	0	CP065183	
prophiGD104-2	MabC	attB-12	1019283–1068838	49,556	73	0	MW584162	–
prophiGD05-1	MabD	attB-10	3676892–3737783	60,892	95	0	MW584169	
prophGD12-2	MabD	attB-10	C1 575019–629478r	54,460	87	0	MW584207	–
prophiGD14-2	MabD	attB-10	C1 769184–714725r	54,460	87	0	JADWWX000000000	
prophiGD17-1	MabD	attB-3	1082962–1134147r	51,224	88	0	MW584165	–
prophiGD25-1	MabE1	attB-4	1888601–1949296	60,696	79	0	MW584148	–
phophiGD04-1	MabE1	attB-4	C2 449800–510231	60,432	78	0	MW584209	–
prophiGD53-1	MabE1	attB-4	C2 573757–513326r	60,432	78	0	CP065033	
prophiGD111-1	MabE1	attB-4	C2 272204–60432	60,432	78	0	JADWYH000000000	
prophiGD54-1	MabE1	attB-4	1852862–1913557	60,696	79	0	MW584189	–
prophiGD68-1	MabE1	attB-4	1673431–1733862	60,432	78	0	MW584157	–
prophiGD102-2	MabE1	attB-4	C1 505277–444582r	60,696	79	0	MW584173	–
prophiGD91-4	MabE2	attB-16	3720180–3778677	58,498	84	0	MW584206	–
prophiGD08-3	MabF	attB-3	C7 175238–227838	52,601	77	0	MW584201	–
prophiGD11-3	MabF	attB-3	C2 175238–229977	54,740	81	0	MW584155	–
prophiGD62-2	MabF	attB-3	C3 66030–120339	54,310	82	0	MW584175	–
prophiGD03-1	MabG	attB-11	C1 1175908–1230728	54,821	85	0	MW584190	–
prophiGD21-3	MabG	attB-11	C1 311838–258956r	52,874	79	0	MW584178	–
prophiGD24-2	MabG	attB-11	C1 260970–313844	52,875	80	0	MW584172	–
prophiGD58-1	MabG	attB-11	C11 101001–157085	56,085	83	0	MW584168	–
prophiGD05-2	MabH	attB-8	3164547–3208415	43,869	68	0	MW584191	–
prophiGD36-2	MabH	attB-8	C2 289068–332653	43,586	70	0	MW584170	–
prophiGD54-2	MabI	attB-9	3370859–3447429	79,047	134	21 (1)	MW584202	M
prophiGD86-1	MabI	attB-17	C7 147871–228663	80,793	144	20	MW584160	M
prophiGD24-3	MabJ	attB-7	C6 248150–303034r	54,885	90	4	MW584159	A
prophiGD43A-4	MabJ	attB-7	2688955–2634523	54,433	91	2	MW584197	A
prophiGD43B-4	MabJ	attB-7	C4 19238–73670	54,433	91	2	CP065278	
prophiGD43A-5	MabK	attB-1	233518–310058	76,541	116	1	MW584167	–
prophiGD43B-2	MabK	attB-1	C7 187106–263646	76,541	116	0	CP065278	
prophiBoletti-1	MabL	attB-10	3445614–3524876	79,288	126	0	AP014547	–
prophiGD43A-6	MabL	attB-10	3745170–3678804	66,367	97	0	MW584174	–
prophiGD43B-1	MabL	attB-10	C1 1282056–1348442	66,367	97	0	CP065278	
prophiGD88-1	MabL	attB-10	C9 21158–87513	66,356	93	0	MW584166	–
prophiGD05-3	MabM	attB-11	3814819–3759328	55,492	76	0	MW584185	–
prophiGD53-3	MabN	attB-13	C1 1154581–1196507	41,918	72	0	MW584183	–
prophiGD62-3	MabN	attB-13	C1 257844–215194r	42,642	68	0	MW584177	–
prophiGD69-1	MabN	attB-13	C12 39118–81768	42,642	68	0	CP065269	
prophiGD108-1	MabN	attB-13	192650–150009r	42,642	68	0	MW584186	–
prophiGD91-3	MabO	attB-14	4808642–4854248	45,607	73	1	MW584187	–
prophiGD51-2	MabP	attB-6	C10 89165–141002	51,838	64	0	MW584195	–
prophiGD79-1	MabQ	attB-4	C2 158082–234229	76,148	108	0	MW584153	–

a Prophages are designated prophiGDXX-1, with GDXX denoting the strain in which it resides and the suffix indicating different prophages in the same strain. Prophages with 100% nucleotide sequence identity are indented related to the identical prophages above them.

b Prophages are grouped into clusters (MabA, MabB, etc.) with closely related prophages in the same cluster. Some clusters are divided into subclusters (MabA1, MabA2, etc.) reflecting sequence relationships.

c attB integration sites are indicated as shown in Fig. 3A.

d Sequence coordinates are shown for completely sequenced genomes. For genomes with WGS sequences, the contig number (e.g., C1, C2 etc.) is shown and the coordinates within that contig. Prophage sequences are similarly oriented and those reverse-complemented are indicated with an “r” suffix.

e Prophage lengths include two copies of the attachment core sites, at the left and right ends of each genome.

f Cluster designations of similarly organized mycobacteriophages (M’phages) are shown, if any; “–“ if not.

g Genome lengths and the number of ORFs are not available (NA) for incompletely assembled prophages.

**TABLE 2:** Plasmids of Mycobacterium abscessus clinical isolates

Name^*a*^	Cluster^*b*^	Length (bp) ^*c*^	ORFs^*d*^	Copy number ^*e*^	Comments^*f*^
pGD08	pA	9547	11	2.9	Mobilizable
pGD42-2	pA	9,547	11	2.6	Mobilizable
pGD18, pGD62-1, pGD69-1, pGD95-1, pGD108A, pGD108B	pB	25,000	37	1.9	Mobilizable
pGD23	pB	25,002	37	1.8	Mobilizable
pGD36-1, pGD47	pB	24,995	37	2.7	Mobilizable
pGD42-1	pB	24,993	38	3.0	Mobilizable
pGD72	pB	24,985	37	1.1	Mobilizable
pGD87	pB	24,994	37	3.0	Mobilizable
pGD22-2, pGD24, pGD34, pGD75, pGD100A, pGD100B	pC	18,117	16	1.9	Mobilizable
pGD39	pC	18,117	16	1.5	Mobilizable
pGD62-2	pC	18,612	17	2.5	Mobilizable
pGD69-2, pGD95-2	pC	18,611	17	1.9	Mobilizable
pGD19	pD	18,605	20	3.1	Mobilizable
pGD45-2	pD	19,406	21	3.1	Mobilizable
pGD85	pD	23,374	26	2.4	Mobilizable
pGD33	pE	25,996	33	1.8	Mobilizable
pGD36-2	pE	24,259	34	5.0	Mobilizable
pGD02	pF	30,963	36	2.2	NA
pGD25-1, pGD54, pGD102-1	pF	31,413	32	2.5	NA
pGD86-1	pF	31,343	32	3.1	NA
pGD25-2	pG	27,424	36	2.5	Mobilizable
pGD45-1	pG	27,427	36	3.9	Mobilizable
pGD86-2	pG	27,424	36	2.3	Mobilizable
pGD102-2	pG	27,425	36	3.9	Mobilizable
pGD58	pH	92,821	122	1.2	Conjugative
pGD13	pSin	21,881	29	1.7	Mobilizable
pGD21-1	pSin	112,633	150	1.1	Conjugative
pGD21-2	pSin	155,609	233	1.3	Linear
pGD22-1	pSin	19,694	21	1.6	Mobilizable
pGD25-3	pSin	23,599	26	5.2	Mobilizable
pGD51	pSin	23,656	27	3.6	Mobilizable
pGD52	pSin	22,216	20	3.2	Mobilizable
pGD104	pSin	96,413	144	1.3	Conjugative
pATCC19977^*g*^	pSin	23,319	29	NA	Mobilizable

a Plasmids are named according to their parent strains (e.g., pGD22). If there is more than one plasmid in a strain, a -1 or -2 suffix is appended. Plasmids with identical sequences are shown in the same row.

b Cluster designation (pA, pB, etc.) is indicated. Singleton plasmids with no close relatives are indicated as pSin.

c Plasmid DNA length is shown in base pairs (bp).

d The predicted numbers of open reading frames (ORFs) are listed.

e Plasmid copy numbers are calculated as the fold-difference between the average number of sequence reads mapping to the plasmid relative to the corresponding genome. If there is more than one plasmid, the average is reported.

f Plasmids are predicted to be mobilizable if they code for a conjugative type relaxase, and conjugative if they contain an ESX operon.

g Plasmid pATCC19977-1 is the same as the previously reported plasmid in this strain (20).

### Diversity of M. abscessus prophages.

The M. abscessus prophages are considerably diverse and can be assorted into 17 clusters (e.g., MabA, MabB, etc.), representing distinct genome sequences (with <35% shared gene content) (Fig. 1C to E, Table 1). Clusters MabA and MabE are sufficiently diverse to warrant division into subclusters (Fig. 1C to E, Table 1). Although most of the prophages are generally not closely related to the thousands of genomically defined M. smegmatis phages, cluster MabI and cluster MabJ prophages are organized similarly to cluster M (24) and A (25) mycobacteriophages, respectively; both share sufficient gene content to warrant inclusion in these clusters (Fig. 1E). Cluster MabA prophages are the most prevalent and are residents of the major clade of closely related M. abscessus subsp. *abscessus* strains (26); however, they are also present in some M. abscessus subsp. *bolletii* and *massilliense* strains (Table 1). Twelve clusters have 6 or fewer members, and four have only a single member (these are assigned to clusters rather than classed as singletons, as there are relatives in the large number of M. abscessus genomes in public databases) (Fig. 1E). The M. abscessus prophages are at least as, if not more, diverse than an equivalent number of M. smegmatis phages (10).

**FIG 2:**
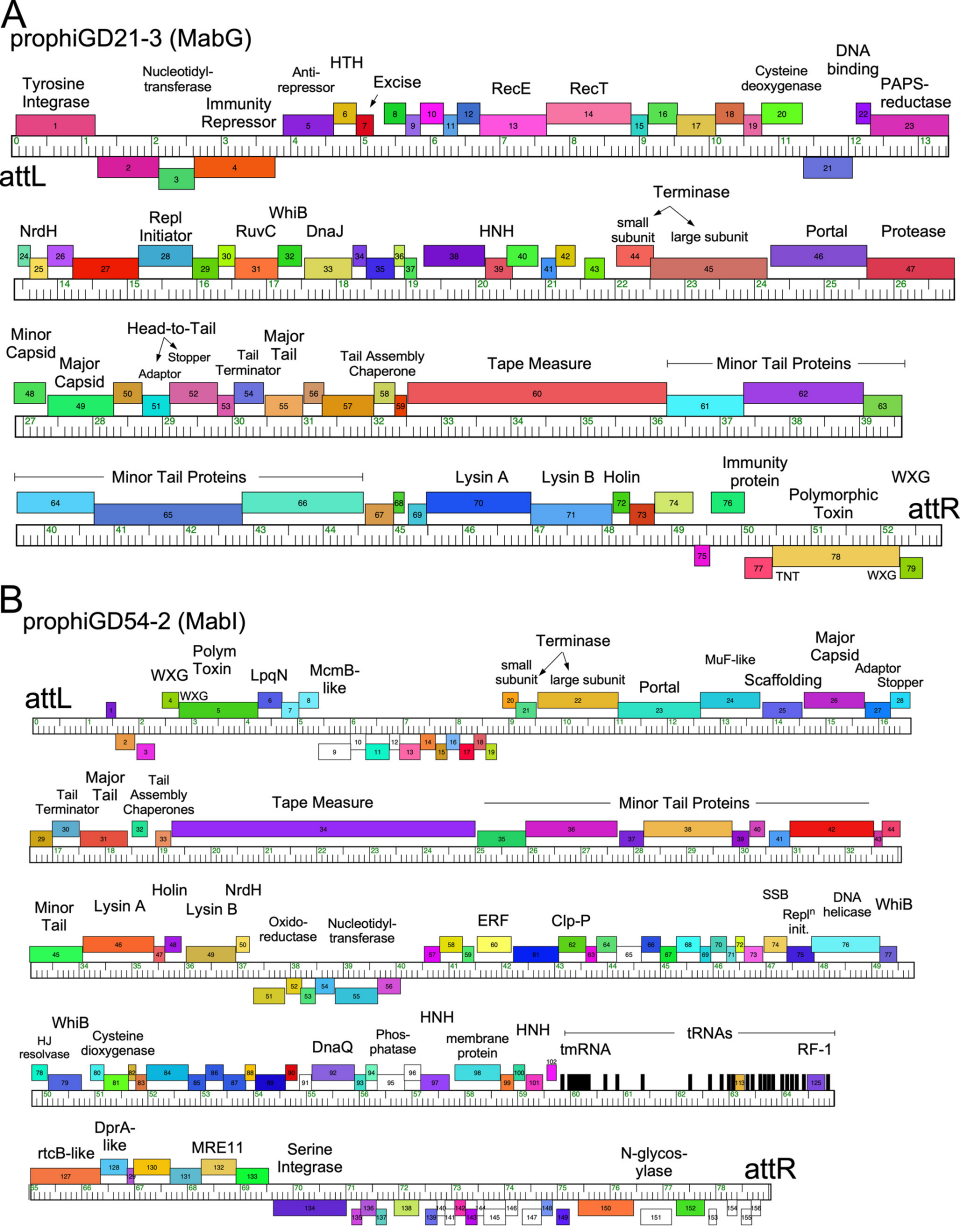
Genome organization of prophiGD21-3 and prophiGD54-2. The organizations of prophageGD21-3 (A) and prophiGD54-2 (B) are shown, with the genes represented as boxes above and below the genome rulers indicating rightward and leftward transcription, respectively. Genes are colored according to the sequence “phamilies” they are assigned to, and tRNAs are represented as black bars. Putative gene functions are indicated above the genes. Genes *5* to *43* of prophiGD21-3 and *57* to *133* of prophiGD54-2 are predicted to be transcribed early in lytic growth, with genes *44* to *76* and *20* to *50*, respectively, coding for virion structure and assembly proteins that are expressed late in lytic growth.

Genomic maps of prophages prophiGD21-3 and prophiGD54-2 (Fig. 2) illustrate some of the interesting and unusual genomic features of these prophages, and detailed genomes of prophages are shown at https://phagesdb.org/documents/categories/14/. ProphiGD21-3, a member of cluster MabG (Fig. 2A), is organized with most of the genes rightward-transcribed, with the notable exceptions of a cassette adjacent to *attR* containing a polymorphic toxin (PT), a corresponding immunity protein (27), and an ESAT-6-like WXG-100 protein (Fig. 2A). The PT contains an N-terminal WGX-100 motif and is likely exported by the host type VII secretion system. The PT contains a C-terminal domain related to the tuberculosis necrotizing toxin (TNT), which facilitates immune evasion by Mycobacterium tuberculosis (28), thus implicating this prophage in success of M. abscessus *in vivo*. These PT-Imm cassettes are common in the prophages but highly varied, as discussed in detail below. A second feature of note is genes *20* and *23*, which are predicted to be expressed early in lytic growth and code for proteins with motifs common to cysteine dioxygenases and phosphoadenosine phosphosulphate (PAPS) reductases, respectively. It is unusual for these to be phage encoded, but PAPS reductase-like proteins are similar to DndC, which participates in phosphorothioate DNA modifications that are common in M. abscessus (29, 30); gp20 is also implicated in cysteine metabolism.

ProphiGD54-2 (cluster MabI) is organized similarly to cluster M mycobacteriophages (24). It integrates with a serine-integrase and codes for an array of 21 tRNA genes and a tmRNA, as well as a release factor (Fig. 2B), suggesting substantial translational reprogramming during lytic growth. However, like prophiGD21-3 (Fig. 2A), prophiGD54-2 also codes for a PT-Imm cassette, although it is located proximal to *attL* (Fig. 2B). The PT also contains an N-terminal WXG-100 motif and has a C-terminal motif distantly related to the AvrE-family of secreted effectors; the Imm protein is a predicted LpqN-like lipoprotien and is likely to be cell wall associated.

### Diversity of M. abscessus plasmids.

Although plasmids are not as prevalent as prophages in these clinical isolates and are only present in ∼50% of the strains, they are also quite diverse (Table 2, Fig. 1F). There are eight clusters (pA to pH) and nine singletons, each without close relatives in this data set, of which three (pGD58, pGD104, and pGD21-1) are large and are not fully assembled (Fig. 1F). The smallest are cluster pA plasmids (9.5 kbp), but the cluster pH and singleton pGD104 plasmids are over 90 kbp. All are present at low copy number, typically fewer than 5 copies/cell on average (Table 2; Fig. 1F). Comparison of these plasmids with the extant publicly available (∼1,500) M. abscessus sequences shows that although some plasmid-borne genes are prevalent, there are few examples of near-full-length sequence matches. Notable exceptions are M. abscessus subsp. *bolletii* plasmid 2 (31) and M. abscessus pJCM30620 (32), which are similar to pGD58 (each with 99% identity spanning 92% coverage), and plasmid *Mycobacterium* sp. djl-10 plasmid djl-10_3 (accession number CP016643.1) that is very similar to pGD25-3. Detailed genome maps of the plasmids are available at https://phagesdb.org/documents/categories/14/.

Plasmids in different clusters share fewer than 35% of their genes, but most code for one of four sequence types of a RepA replication protein, the exceptions being the large plasmids pGD58 (cluster pH), pGD21-1, and pGD104, for which no RepA was identified. RepA sequences of pA, pC, pD, pE, and pF plasmids are sufficiently similar (>64% pairwise amino acid [aa] identity) that they likely form a single incompatibility (Inc) group (IncMabI) (Fig. 1F). Clusters pB, pG, and singletons pATCC19977, pGD13, pGD22-1, pGD51, and pGD52 have a second group of related RepA proteins (>75% pairwise aa identity), potentially forming a second Inc group (IncMabII), and although 10 strains have two plasmids, none have two plasmids of the same Inc type. GD25 has three plasmids and singleton pGD25-3 likely represents a third Inc group (IncMabIII), although it shares 78% aa identity with IncMabII plasmid pGD25-2 (MabG). GD21 has two plasmids, pGD21-1 and -2, the latter of which is linear and represents a fourth Inc group (IncMabIV) (Fig. 1F).

### Prophage locations and prophage integration.

**FIG 3:**
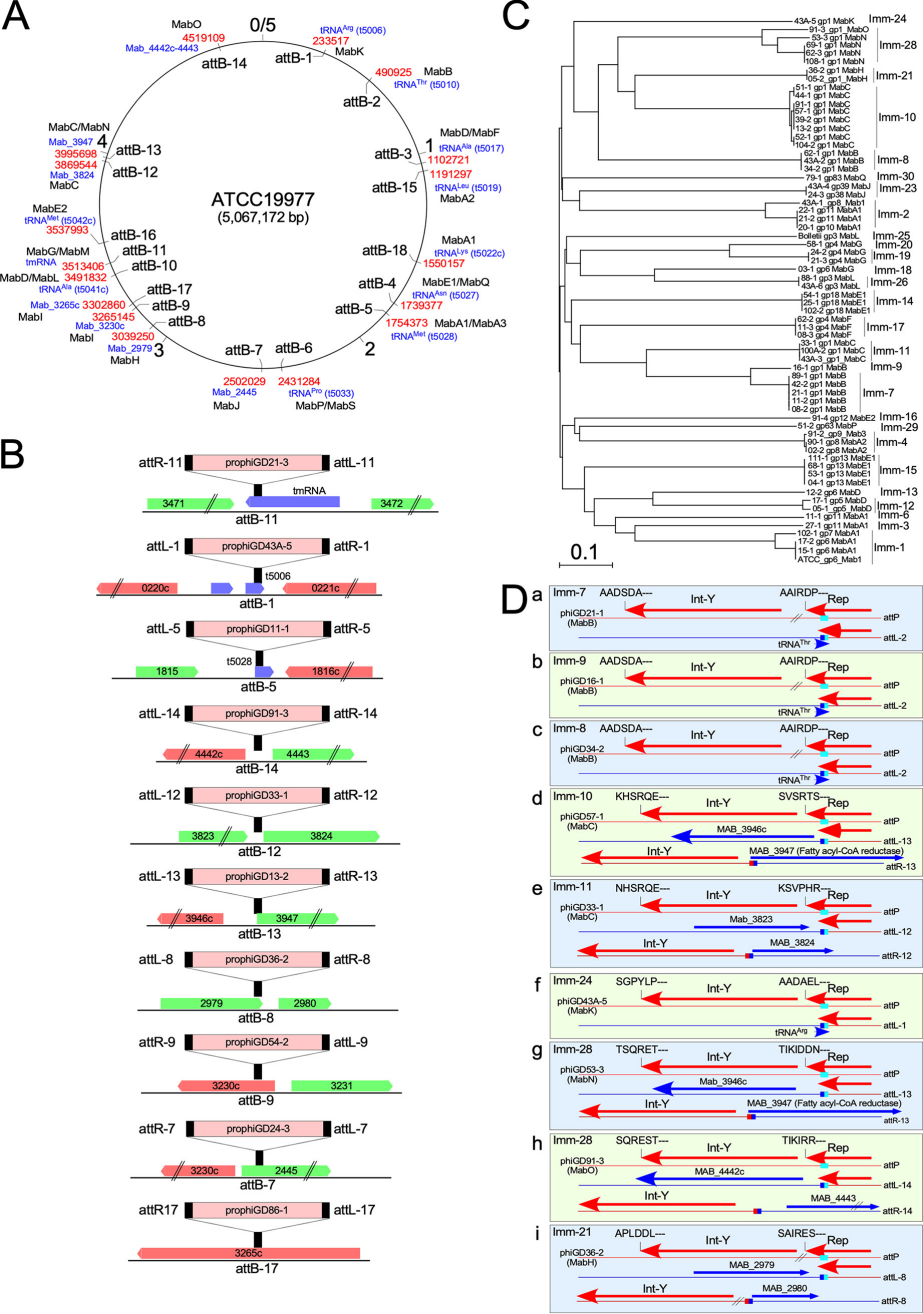
M. abscessus prophage integration and immunity. (A) Location of *attB* sites in the M. abscessus genome. The 5-Mbp M. abscessus ATCC 19977 circular genome is represented, with the location of the 18 *attB* sites (attB-1 to attB-18) indicated inside the circle. Outside the circle the coordinate of the site in ATCC 19977 is shown in red, the associated M. abscessus ATCC 19977 gene name is shown in blue, and the prophage clusters using each *attB* are shown in black. (B) *attB* locations and consequences of integration. Ten examples of *attB* site locations are shown (black bars) relative to the M. abscessus ATCC 19977 genes for reference; rightward and leftward transcribed genes are shown as green and red boxes, respectively, with their ATCC 19977 gene number. An integrated prophage example is shown for each *attB* site, with the corresponding *attL* and *attR* sites shown to reflect the orientation of integration. The *attB* sites not shown (attB-2, attB-3, attB-4, attB-6, attB-10, attB15, attB-16, and attB-18) all overlap the 3′ end of a host tRNA gene, as illustrated for attB-1. Systems using Int-Y or Int-S are indicated. (C) Superinfection immunity groups of M. abscessus prophages. Phylogenetic relationship of putative prophage repressors are shown with designation of immunity groups (Imm-1, Imm-2, etc). (D) Integration-dependent immunity systems. Examples are shown of nine distinct immunity groups (boxes a to i), each of which uses an integration-dependent immunity system in which the *attP* site (aqua-colored box) is located within the repressor gene (Rep). Each code for a tyrosine family integrase (Int-Y) either immediately downstream of Rep or separated by 3 to 5 genes (//). Integration results in a truncated but active form of the repressor due to a closely linked translation stop codon at *attL* (blue/aqua box). The six C-terminal amino acid residues of Int and Rep are shown, showing that many of the *rep* and *int* genes have ssrA-like degradation tags (XXXAA-C-term), whereas others do not and presumably use alternative degradation systems.

All of the prophages are chromosomally integrated, and many are expected to impact host physiology; no plasmidial prophages were identified (33). They are inserted at 18 different positions and are distributed broadly around the M. abscessus genome (Fig. 3A); the number and variety of *attB* sites is greater than those used by 1,800 sequenced phages of M. smegmatis (34). Phages in most of the clusters use a tyrosine integrase (Int-Y), with the exceptions of clusters MabI and MabJ, which both use serine integrases (Int-S) (Fig. 1E). Of the 15 *attB* sites used by Int-Y, 10 overlap host tRNA genes, a common organization for these integration systems (35); however, 5 do not (Fig. 3A). MabG and MabM phages use an *attB* site (attB-11) located within the host tmRNA gene (Fig. 3B). The common core sequences (shared by *attB* and *attP*) are typically 25 to 76 bp for the tRNA-*attB* sites (Table S1), with the phage-derived sequences reconstructing the 3′ end of the host tRNA gene (e.g., attB-1; Fig. 3B); the exceptions are the MabA1 phages that unusually reconstruct the 5′ end of the tRNA^Met^ gene at *attB* (e.g., attB-5; Fig. 3B). For all of these, no host genes are lost upon integration, although the tRNA^Met^ gene (Mab_t5028) must be expressed from a phage promoter following MabA1 phage integration at attB-5 (Fig. 3A and B). Int-Y phages integrating at the four non-tRNA *attB* sites (attB-14, attB-12, attB-13, and attB-8) typically have shorter core sequences (3 to 25 bp), but the consequences of integration are more complex (Fig. 3B). At attB-14, the integration is intergenic (MAB_4442-4443) and flanking gene expression is likely unaltered following integration. However, at attB-12 and attB-13, the common core overlaps the ribosome-binding site and translational start site of MAB_3824 and MAB_3947 (fatty acyl-CoA reductase), respectively, such that transcription of these genes must originate within the prophages (Fig. 3B). The attB-8 site is within the 3′ end of MAB_2979, with the crossover site positioned seven codons from the translation stop codon (Fig. 3B), and, although integration results in replacement of the C-terminal seven amino acids with eight prophage-derived residues, the protein likely retains functionality (Fig. S1).

Three *attB* sites (attB-9, attB-7, and attB-17) are used by Int-S systems and have characteristically short common core sequences (5 to 8 bp) (36, 37). All integrate within open reading frames which they disrupt, as described similarly for Bxb1 integration into the *groEL1* gene of M. smegmatis (36, 38). The cluster MabJ phages integrate at attB-7 located within MAB_2445, which encodes an AraC-like regulator, with potential for wholesale changes in host gene expression. attB-9 and attB-17 are both used by MabI phages and are located within MAB_3230 and MAB_3265, respectively (Fig. 3B). Mab_3230 contains a SnoaL_4 domain and is related to an oxidoreductase of *Streptomyces* (39). MAB_3265 encodes a dienelactone hydrolase family protein, although its specific role is not known.

### Superinfection immunity and integration-dependent immunity.

There is considerable variation in the sequences of immunity repressors, including within clusters of otherwise closely related prophages (Fig. 3C, Table S1). At least 30 distinct immunity groups are predicted, reflecting a broad capacity to influence phage infection profiles by repressor-mediated superinfection immunity (Fig. 3C, Table S2). With the exceptions of the cluster MabI and MabJ phages, the repressors are divergently transcribed from putative *cro*-like genes and closely linked to the *int*; they vary considerably in length and sequence, but most contain putative DNA-binding motifs. In the cluster MabJ phages, the repressor is distal from the integrase, reflecting the organization common to cluster A mycobacteriophages. The repressor location in the cluster MabI is unclear.

Nine of these prophages, corresponding to six *attB* sites (attB-1, attB-2, attB-8, attB-12, attB-13, and att-14), use integration-dependent immunity systems (40). These systems are unusual in that *attP* is located within the repressor gene such that the viral- and prophage-encoded gene products differ at their C termini. The virally encoded repressor gene product typically has a C-terminal ssrA-like degradation tag and does not confer immunity, and integration is required for removal of the degradation tag and expression of a functional repressor (40). Clusters MabB, MabC, MabH, MabK, MabN, and MabO all have *attP* within their repressor genes and integrative recombination leads to a 20 to 35 residue shorter gene product truncated at its C terminus due to a translation stop codon at *attL* (Fig. 3D, Fig. S1). For the MabC, MabK, MabN, and MabO phages (using attB-1, attB-12, attB-13, and attB-14), the stop codon is formed by juxtaposition of the first base of bacterial sequence to the phage sequence at *attL*. In the MabB phages, seven amino acids are added from the bacterially derived sequence (Fig. S1). For MabB and MabK phages, the *attB* site overlaps the 3′ ends of tRNA genes such that the tRNA is transcribed toward the truncated repressor (Fig. 3D).

The Imm-7, Imm-9, Imm-8, and Imm-24 virally encoded repressors have C-terminal sequences (-AA) consistent with degradation by the ssrA system and, with the exception of Imm-24, their integrases also have -AA C termini (Fig. 3D), similar to the previously described systems in mycobacteriophages (40). The MabC, MabN, MabO, and MabH repressors do not have ssrA-like tags and presumably use other signals for degradation, similar to phage BPs (40). The integrases of MabC, MabK, MabN, MabO, and MabH also do not have ssrA-tags (Fig. 3D).

### Prophage-encoded polymorphic toxin-Imm systems.

**FIG 4:**
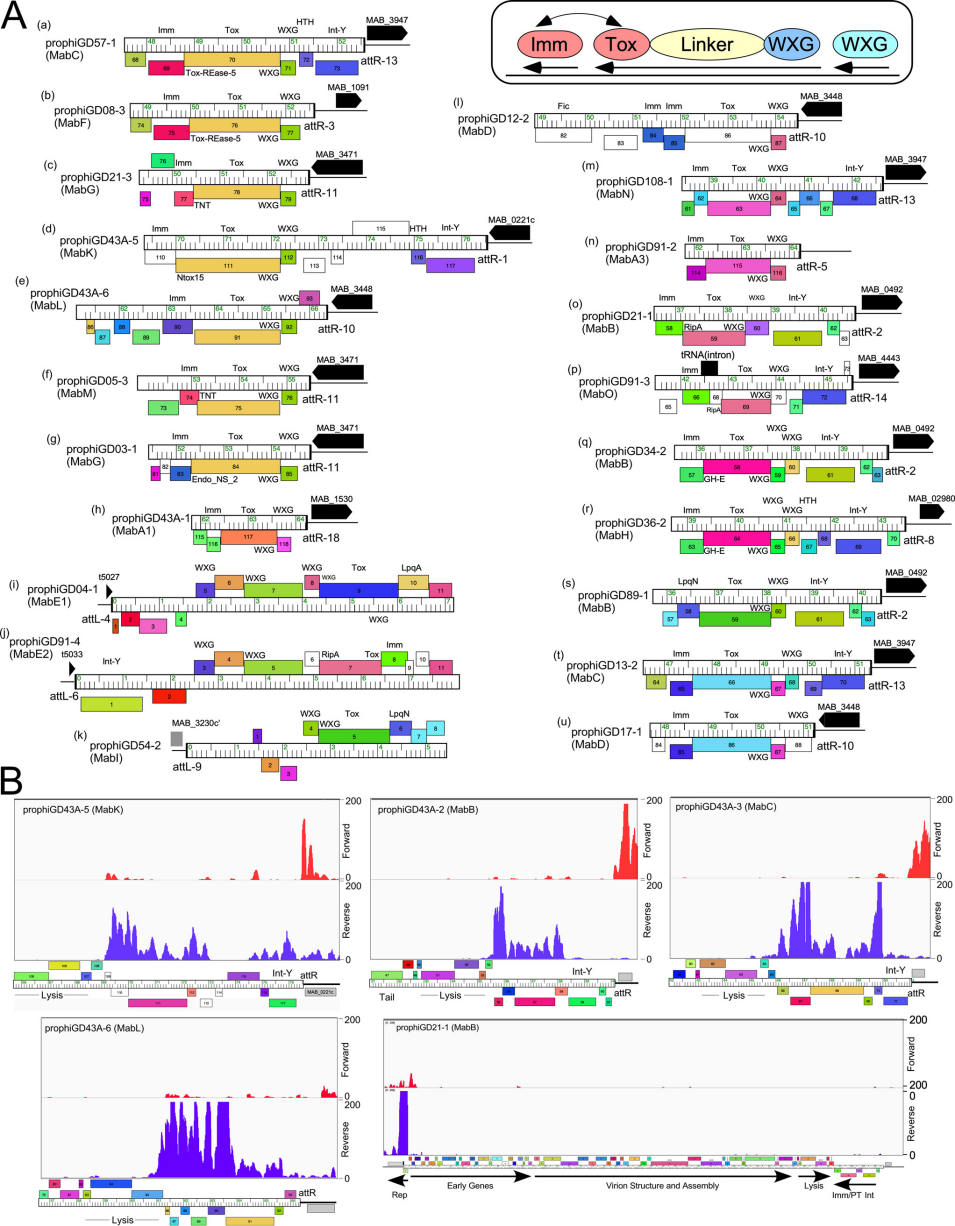
Polymorphic toxin-immunity regions of M. abscessus prophages. (A) Fifty of the complete M. abscessus prophages identified carrying a region coding for a polymorphic toxin, immunity protein, and WXG-100 genes organized into 21 distinct arrangements (labeled a to u). In each arrangement these genes are close to either an *attR* (a to h, l to u) or *attL* (i to k) attachment junction (designated according to the *attB* site used; see Fig. 2A, Table S3) and phages genes are shown as colored boxes above or below genome rulers reflecting rightward and leftward transcription, respectively; black arrows indicate a host gene adjacent to the attachment, designated with the corresponding gene number in M. abscessus ATCC 19977. The genomes are aligned by 5′ end of the toxin gene (a to h, l to u) where transcribed leftward inside *attR*, and similarly for the three configurations adjacent to *attL* (i to k), where the genes are transcribed rightward. Genes are colored according to their designated assignment into groups of related proteins (phamilies). All of the polymorphic toxin genes have an N-terminal WXG-100 (WXG) motif common to the type VII secretion system but have variable C termini. A schematic representation is shown in the box at top right indicating the organization of the polymorphic toxin domains and the proposed interaction between the toxin and a protective immunity protein. (B) Expression of the PT-Imm loci. RNAseq profiles for prophiGD43A-5, prophiGD43A-2, prophiGD43A-3, and prophiGD43A-6 show lysogenic expression of the PT-Imm loci; most of the rest of the prophages are transcriptionally silent. RNA was prepared from M. abscessus strain GD43, and only sequence reads mapping uniquely are shown. Also shown is a profile of the entire prophiGD21-1 prophage, in which only the repressor is expressed. RNA was prepared from M. abscessus strain GD21 and RNAseq reads mapping to forward (red) and reverse (purple) strands are shown.

The presence of PT-Imm cassettes in prophiGD21-3 and prophiD54-2 was noted above (Fig. 2), but related cassettes are prevalent in these prophage genomes. Prophages in 14 clusters code for a remarkably diverse set of PT-Imm systems, all implicated in bacterial virulence (41) (Fig. 4A, Table S3). These systems code for a large (∼50 kDa) member of the polymorphic toxin (PT) family, and an immunity protein (Imm) that protects from toxicity (41). All of the prophage-encoded toxins include an N-terminal WXG-100 motif targeting the PT for export by the type VII secretion system (TSS), together with a small ESAT6-like protein with a WXG-100 motif that likely forms a heterodimer to promote PT export (Fig. 4A). The variation among the prophage-encoded PTs is considerable, including at least 10 different sequence groups of the PTs, with additional diversity among their C-terminal regions. For example, prophages prophiGD57-1, prophiGD08-3, prophiGD21-3, prophiGD43A-5, prophiGD43A-6, prophiGD05-3, and prophiGD03-1 code for related PTs, but the C-terminal regions code for different motifs, including Tox-REase-5, tuberculosis necrotizing toxin (TNT), Endo-NS2, and Ntox-15 motifs (Fig. 4A, a to g). The putative Imm proteins immediately downstream of the PTs are also highly diverse and are predicted to interact directly with the toxin (42), and likely coevolve with the PT C-terminal domain (Fig. 4A). Thus, although there are seven different configurations with a toxin related to that in prophiGD57-1 (Fig. 4A), the four different putative Imm proteins correspond to the C-terminal variation of the toxin (Fig. 4A). We note that several of the putative Imm proteins are predicted lipoproteins (Fig. 4A, i, k, and s). Secretion of the PT likely utilizes either the M. abscessus Esx-3 or Esx-4 type VII secretion systems, both of which are important for growth *in vivo* (43, 44). These prophage-encoded PT-Imm systems are predicted to contribute to M. abscessus *in vivo* growth and infection via multiple mechanisms.

All of these PT-Imm systems are encoded close to the attachment junctions and adjacent to bacterial genes (Fig. 4A), a common location for prophage-expressed genes among mycobacteriophages (13, 15). Transcriptome sequencing (RNAseq) shows that most prophage genes are transcriptionally silent, but the PT-Imm systems are expressed in several lysogens with transcription initiation originating from prophage promoters (Fig. 4B). The Imm genes are expressed at higher levels than the PT genes, presumably to optimize immunity from the PT prior to export (Fig. 4B). This is in contrast to the MuF-related toxins within the virion structural genes of several Escherichia coli phages, which are secreted by type VI systems (27, 45). We note, however, that PT-Imm expression is not observed in all lysogenic strains, as shown by prophiGD21-1, in which the repressor is the sole lysogenically expressed gene product (Fig. 4B). It is plausible that some PT-Imm systems are expressed only in host cells.

### Prophage-encoded toxin-antitoxin systems.

**FIG 5:**
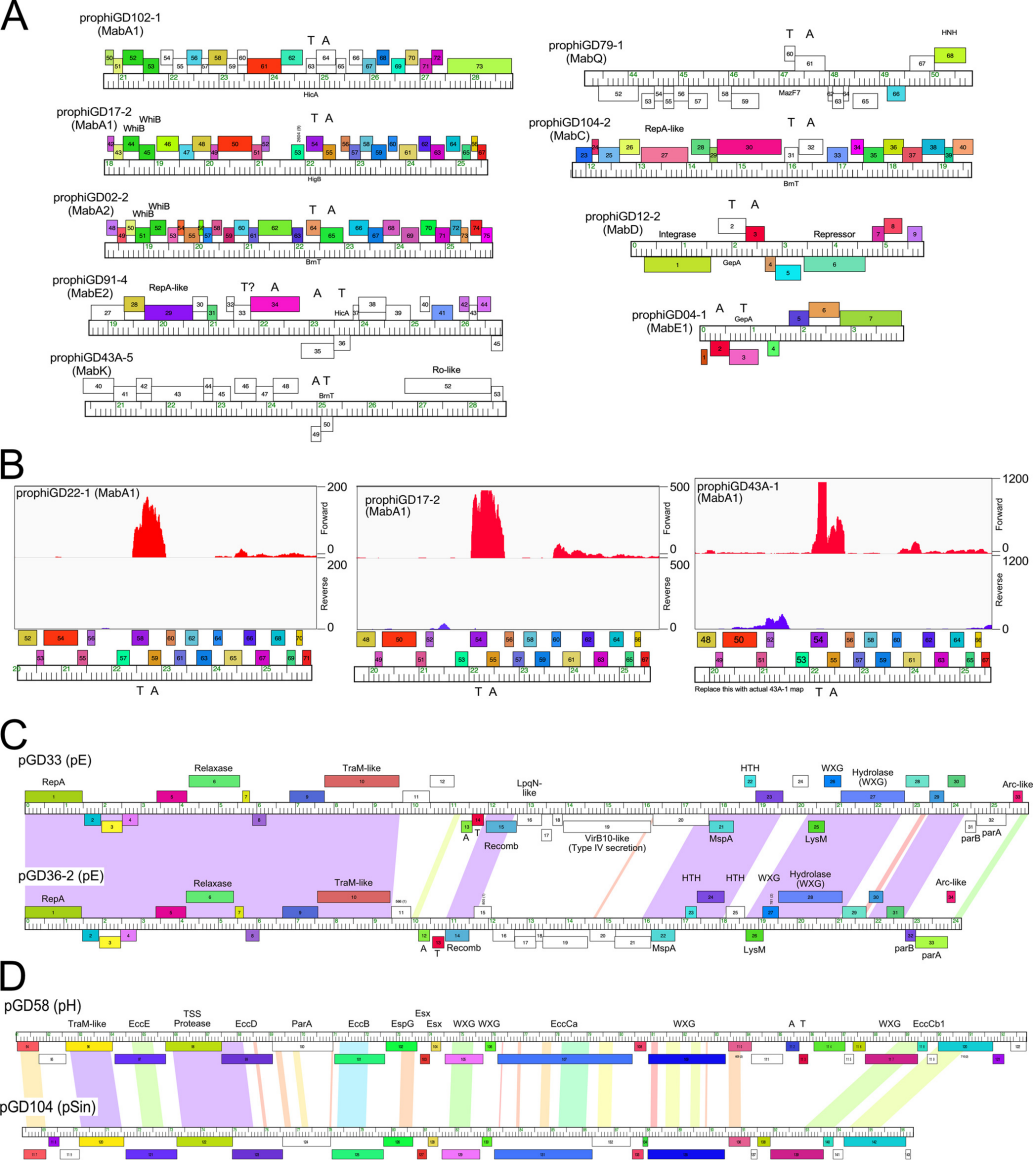
Prophage- and plasmid-borne genes. (A) Toxin-antitoxin (TA) systems in M. abscessus prophages. Genome maps for prophage segments are displayed as described in Fig. 2, with the location of TA genes indicated. (B) RNAseq profiles showing lysogenic expression of TA modules. RNA was isolated from GD22, GD17, and GD43A and strand-specific reads were mapped to prophage regions (prophiGD22-1, prophiGD17-2, and prophiGD43A-1). Read mapping to forward (red) and reverse (purple) strands are indicated. (C) Organization of cluster pE plasmids pGD33 and pGD36-2. Both plasmids encode plasmid replication (RepA, ParA, and ParB), site-specific recombination (Recomb), and mobilization (Relaxase, TraM-like) functions, as well as type VII secretion genes (WXG), lipoprotein (LpqN), porin (MspA), and TA systems. pGD33 also codes for a VirB10-like factor. (D) Genome organization of the Esx loci of large plasmids pGD58 and pGD104; only the rightmost 30 kbp of the genomes are shown. Components of the TSS Esx systems are indicated. Pairwise nucleotide sequence similarity is displayed by spectrum color shading between the genomes, with violet as most similar and red as least similar. Genes are shown as boxes either above or below the genome, indicating rightward and leftward transcription, respectively. Gene boxes are colored according to gene phamilies in which they are assigned.

Prophages can encode and express multiple functions other than repressor-mediated immunity that prevent phage infection, often with considerable specificity and against genomically unrelated phages (3, 5). Among these are toxin-antitoxin (TA) systems and several are located in *att*-linked defense loci of mycobacteriophages and are prophage expressed (3). Nineteen M. abscessus prophages code for at least nine different TA systems, although only two (in prophiGD12-2 and prophiGD04-1) are proximal to an attachment site (Fig. 5A). The others are located within early lytic genes but often transcribed on the opposite strand (e.g., prophiGD79-1, prophiGD91-4, prophiGD43A-5, and prophiGD12-2) (Fig. 5A). RNAseq of several lysogens carrying MabA1 phages shows that the TA pair is strongly transcribed, contrasting with the flanking phage (Fig. 5B). These genes are thus implicated in influencing bacterial physiology and likely promote defense against viral infection.

### Potential roles of M. abscessus plasmids.

The M. abscessus plasmid repertoire is diverse and replete with functions predicted to influence bacterial physiology, including antibiotic resistance, phage defense, and virulence. Most of the plasmids are likely mobilizable and code for conjugative-type relaxases, perhaps using the host TSS systems for mobilization that are implicated in distributive conjugation (46). We note that clusters pC, pD, and pE plasmids also code for several proteins with WXG-100 domains that are likely also exported through the TSS system, as well as toxin-antitoxin and abi systems (47) implicated in viral defense (Fig. 5C). Abi genes (47) are present in clusters pA, pB, pC, pD, pF, and the singletons pATCC19977, pGD25-3, and pGD104, and TA systems are in plasmids in clusters pD, pE, pH, and singletons pGD104 and pGD21-2. However, we note that of the 28 strains that are not infected by phages, 19 are plasmid free, and the overall phage susceptibility profiles are likely determined by complex combinations of prophage, plasmid, and bacterially encoded functions (17).There are also a variety of genes associated with transport systems, including the MmpL proteins (coded by pB plasmids), MFS-like transporters, and several metal resistance and iron regulators, specifically. These strains are resistant to many different antibiotics and the plasmids are strongly implicated in these resistance phenotypes.

The large (>92 kbp) plasmids (pGD58, pGD104, pGD21-1, and pGD21-2; Table 2) are notable in that they have large (25 to 30 kbp) ESX regions coding for type VII secretion systems that are implicated in conjugative plasmid transfer (Fig. 5D); these ESX systems are similar to that in *M. bolletii* 50594 plasmid 2, designated ESX-P cluster 3 (48). Related plasmids are reported to be quite widespread (49), but are not highly prevalent in M. abscessus strains; pGD58 and pGD104 each have only ∼20 closely related plasmids in over 1,500 sequenced M. abscessus strains (50). The three strains carrying these large plasmids are all M. abscessus subsp. *massiliense*, two of which have smooth morphotypes, suggesting that abundant surface GPLs do not interfere with plasmid transfer by conjugation.

## DISCUSSION

M. abscessus is an important emergent pathogen and widespread antibiotic resistance presents substantial clinical challenges. Elucidating its pathogenic capacity is complicated by its genetic variability, much of which could be driven by its expansive mobilome of prophages and plasmids, many of which code for genes predicted to influence survival and growth *in vivo* as well as antibiotic- and phage-resistance profiles (Fig. 4, Fig. 5). Defining these strain differences and their pathogenic behaviors is of considerable importance (17). Most studies of M. abscessus have focused on type-strains such as ATCC 19977, but this strain is poorly representative of the pathogenic potential and physiology of most clinical strains, whose mobilomes are revealed to be highly diverse, with individual strains having different properties depending on the variety of prophages and plasmids they carry. Understanding clinical responses to M. abscessus infection will require a much broader understanding of these strain differences and their phenotypic consequences.

The widespread antibiotic resistance of M. abscessus clinical isolates is a substantial impediment to genetic manipulation, as it greatly limits the use of selectable markers for transformation. The diverse prophage and plasmid repertoires offer a multitude of opportunities for advancing the genetic systems. For example, the numerous superinfection immunity systems are a resource for use as genetically selectable markers that circumvent the use of antibiotics (51). Several of the prophages have been propagated lytically and it is likely that many more can be (17, 52). For each of these, a cloned repressor gene can be adapted as a selectable gene using lytic phage derivatives as selective agents. We note that for the integration-dependent immunity systems (40), it is critical that the truncated-but-active prophage-encoded repressor must be used, not the inactive virally encoded form.

There are relatively few plasmid replicons available for vector development for M. abscessus. The plasmids described here represent at least four incompatibility groups (Fig. 1F), each of which could be used to develop low-copy-number extrachromosomal vectors for combinatorial use. There is also considerable potential for construction of additional integration-proficient plasmid vectors taking advantage of the abundance of newly identified *attB* sites (Fig. 3A). We note that the commonly used integrative vectors based on mycobacteriophage L5 (53) use a conserved *attB* site overlapping M. abscessus tRNA^Gly^ gene (t5027), which is not occupied by any of the prophages described here (Fig. 3B), and therefore should be broadly applicable.

## MATERIALS AND METHODS

### Bacterial strains and media.

M. smegmatis mc^2^155 was grown as previously described (14). M. abscessus strains were grown in 10 ml of 7H9 medium with oleic acid-albumin-dextrose-catalase (OADC) and 1 mM CaCl_2_ for ∼72 h at 37°C with shaking. For some M. abscessus strains, several individual isolates were recovered either at different times or different morphotypes, including strains GD54, GD35, and GD64, which were designated GD54H, GD35B, and GD64A, respectively. For some isolates, both rough and smooth colony morphotypes were recovered, and designated accordingly (e.g., GD68A, GD68B). GD43A and B have different numbers of prophages in them and they are therefore treated as separate strains. Bacterial DNA was prepped from 1 ml of log-phase culture using standard phenol-choloroform-isoamyl alcohol extraction and ethanol precipitation. Phage DNAs were isolated using similar methods as reported previously (9).

### Genomics.

Genomic DNAs were prepared for sequencing using NEB Ultra II FS kits and then pooled and run on an Illumina MiSeq using v3 reagent kits to generate 300-base paired-end reads. In some cases, Oxford Nanopore sequencing libraries were also constructed from genomic DNA using Rapid Sequencing Barcoding kits, and then pooled and run on a MinION device using FLO-MIN106D flowcells. Illumina reads for each strain were trimmed and quality-controlled using Skewer (54). Trimmed Illumina reads were then assembled using Unicycler (55), incorporating Nanopore reads when available.

In the case of complete genomes, assemblies were viewed, stitched, corrected, and finalized using Consed version 29 (56, 57). GraphMap (58) was used to align long Nanopore reads to provisional assemblies and resolve repetitive regions. The first base and orientation of each complete circular chromosome was chosen to match those of the ATCC 19977 strain and/or to align with the first base of the *dnaA* gene.

### Prophage and plasmid identification.

Prophages were detected initially by searches using PHASTER (20) followed by careful manual inspection. PHASTER often identifies potential regions with prophages but does not accurately identify attachment junctions. Precise prophage positions were determined by genome comparisons with strains lacking those prophages, and identifying the short repeated sequences corresponding to the common core at the *attL* and *attR* sites. Related copies of prophages were identified by extensive sequence searches and genome comparisons. Each prophage sequence was extracted, including the common core sequence at both ends of the prophage genome. Prophages were designated according to the strain in which they reside, i.e., prophiGDXX-1, with suffixes used to denote multiple prophages in the same genome.

Potential plasmids were identified primarily as small circularized contigs in genome assemblies, although one linear plasmid was also identified. These contigs were manually inspected to ensure they were valid, complete, and not contaminants. Complete circular plasmids were oriented and cut so that base 1 was the first base of a predicted *repA* gene whenever possible.

### Other bioinformatics.

Phylogenies were constructed using neighbor joining with ClustalX and NJPlot, or were created using CSI Phylogeny 1.4, a SNP-based concatenated alignment, available on the DTU server (https://cge.cbs.dtu.dk/services/CSIPhylogeny/) (59). A prophage network phylogeny based on gene content was constructed using Splitstree (60) similarly to as was described previously (9). Phamerator (61) databases “Actino_prophage_15” and “Mycobacterium_prophages_5” were constructed for comparative genomic analyses.

### RNAseq.

Total RNA was isolated from logarithmically growing M. abscessus cells. Removal of DNA was completed using a Turbo-DNase-Free kit (Ambion) according to the manufacturer’s instructions. The depletion of rRNA was completed using QIAseq FastSelect (Qiagen). The libraries were constructed using the NEBNext Ultra RNA library kit (New England BioLabs) and verified using a BioAnalyzer. The libraries were multiplexed and 4 were run on an Illumina MiSeq for each run. Analysis of the data was as described previously (62). Only unique reads were mapped to each genome set. All RNAseq data have been deposited in Gene Expression Omnibus (GEO) repository (GSE161710).

### Data availability.

The data that support the RNAseq findings of this study have been deposited in Gene Expression Omnibus (GEO) with number GSE161710. The completed and WGS genome sequencing data for M. abscessus clinical isolates, including plasmids and prophages, are available in GenBank, and a complete list of accession and project numbers are provided in the accompanying manuscript (17).

10.1128/mBio.03441-20.1 TABLE S1 *attB* sites in M. abscessus Download Table S1, PDF file, 0.06 MB. Copyright © 2021 Dedrick et al. 2021 Dedrick et al. https://creativecommons.org/licenses/by/4.0/ This content is distributed under the terms of the Creative Commons Attribution 4.0 International license.

10.1128/mBio.03441-20.2 TABLE S2 Predicted M. abscessus prophage immunity groups Download Table S2, PDF file, 0.05 MB. Copyright © 2021 Dedrick et al. 2021 Dedrick et al. https://creativecommons.org/licenses/by/4.0/ This content is distributed under the terms of the Creative Commons Attribution 4.0 International license.

10.1128/mBio.03441-20.3 TABLE S3 Types of polymorphic toxin-immunity configurations Download Table S3, PDF file, 0.02 MB. Copyright © 2021 Dedrick et al. 2021 Dedrick et al. https://creativecommons.org/licenses/by/4.0/ This content is distributed under the terms of the Creative Commons Attribution 4.0 International license.

10.1128/mBio.03441-20.4 FIG S1 Integration-dependent immunity systems. Sequences are shown for phage (phiGDxx) and prophage (prophiGDxx) sequences at the 3′ ends of the repressor genes; if the phage sequence is reconstructed from the prophage sequence, it is designated in apostrophes. The amino acid sequence of the repressor is highlighted in green, and tRNA genes in grey shading. Common core sequences are underlined. Integrative recombination occurs within the repressor coding region, giving rise to a C-terminally truncated repressor expressed from the prophage. For MabH with example of prophiGD36-2, we show the sequences of *attL* and *attR* and deduced *attB* and *attP* sequences, highlighting changes in DNA and amino acid sequences. Download FIG S1, PDF file, 0.07 MB. Copyright © 2021 Dedrick et al. 2021 Dedrick et al. https://creativecommons.org/licenses/by/4.0/ This content is distributed under the terms of the Creative Commons Attribution 4.0 International license.

## References

[1] Hyman P, Abedon ST. 2010. Bacteriophage host range and bacterial resistance. Adv Appl Microbiol 70:217–248. doi:10.1016/S0065-2164(10)70007-1. 20359459

[2] Bondy-Denomy J, Qian J, Westra ER, Buckling A, Guttman DS, Davidson AR, Maxwell KL. 2016. Prophages mediate defense against phage infection through diverse mechanisms. ISME J 10:2854–2866. doi:10.1038/ismej.2016.79. 27258950 PMC5148200

[3] Dedrick RM, Jacobs-Sera D, Bustamante CA, Garlena RA, Mavrich TN, Pope WH, Reyes JC, Russell DA, Adair T, Alvey R, Bonilla JA, Bricker JS, Brown BR, Byrnes D, Cresawn SG, Davis WB, Dickson LA, Edgington NP, Findley AM, Golebiewska U, Grose JH, Hayes CF, Hughes LE, Hutchison KW, Isern S, Johnson AA, Kenna MA, Klyczek KK, Mageeney CM, Michael SF, Molloy SD, Montgomery MT, Neitzel J, Page ST, Pizzorno MC, Poxleitner MK, Rinehart CA, Robinson CJ, Rubin MR, Teyim JN, Vazquez E, Ware VC, Washington J, Hatfull GF. 2017. Prophage-mediated defence against viral attack and viral counter-defence. Nat Microbiol 2:16251. doi:10.1038/nmicrobiol.2016.251. 28067906 PMC5508108

[4] Fineran PC, Blower TR, Foulds IJ, Humphreys DP, Lilley KS, Salmond GP. 2009. The phage abortive infection system, ToxIN, functions as a protein-RNA toxin-antitoxin pair. Proc Natl Acad Sci U S A 106:894–899. doi:10.1073/pnas.0808832106. 19124776 PMC2630095

[5] Dy RL, Richter C, Salmond GP, Fineran PC. 2014. Remarkable mechanisms in microbes to resist phage infections. Annu Rev Virol 1:307–331. doi:10.1146/annurev-virology-031413-085500. 26958724

[6] Hampton HG, Watson BNJ, Fineran PC. 2020. The arms race between bacteria and their phage foes. Nature 577:327–336. doi:10.1038/s41586-019-1894-8. 31942051

[7] Luong T, Salabarria AC, Roach DR. 2020. Phage therapy in the resistance era: where do we stand and where are we going? Clin Ther 42:1659–1680. doi:10.1016/j.clinthera.2020.07.014. 32883528

[8] Hatfull GF. 2020. Actinobacteriophages: genomics, dynamics, and applications. Annu Rev Virol 7:37–61. doi:10.1146/annurev-virology-122019-070009. 32991269 PMC8010332

[9] Pope WH, Bowman CA, Russell DA, Jacobs-Sera D, Asai DJ, Cresawn SG, Jacobs WR, Hendrix RW, Lawrence JG, Hatfull GF, Science Education Alliance Phage Hunters Advancing Genomics and Evolutionary Science, Phage Hunters Integrating Research and Education, Mycobacterial Genetics Course. 2015. Whole genome comparison of a large collection of mycobacteriophages reveals a continuum of phage genetic diversity. Elife 4:e06416. doi:10.7554/eLife.06416. 25919952 PMC4408529

[10] Hatfull GF, Jacobs-Sera D, Lawrence JG, Pope WH, Russell DA, Ko CC, Weber RJ, Patel MC, Germane KL, Edgar RH, Hoyte NN, Bowman CA, Tantoco AT, Paladin EC, Myers MS, Smith AL, Grace MS, Pham TT, O'Brien MB, Vogelsberger AM, Hryckowian AJ, Wynalek JL, Donis-Keller H, Bogel MW, Peebles CL, Cresawn SG, Hendrix RW. 2010. Comparative genomic analysis of 60 mycobacteriophage genomes: genome clustering, gene acquisition, and gene size. J Mol Biol 397:119–143. doi:10.1016/j.jmb.2010.01.011. 20064525 PMC2830324

[11] Russell DA, Hatfull GF. 2017. PhagesDB: the actinobacteriophage database. Bioinformatics 33:784–786. doi:10.1093/bioinformatics/btw711. 28365761 PMC5860397

[12] Montgomery MT, Guerrero Bustamante CA, Dedrick RM, Jacobs-Sera D, Hatfull GF. 2019. Yet more evidence of collusion: a new viral defense system encoded by Gordonia phage CarolAnn. mBio 10:e02417-18. doi:10.1128/mBio.02417-18. 30890601 PMC6426606

[13] Gentile GM, Wetzel KS, Dedrick RM, Montgomery MT, Garlena RA, Jacobs-Sera D, Hatfull GF. 2019. More evidence of collusion: a new prophage-mediated viral defense system encoded by mycobacteriophage Sbash. mBio 10:e00196-19. doi:10.1128/mBio.00196-19. 30890613 PMC6426596

[14] Jacobs-Sera D, Marinelli LJ, Bowman C, Broussard GW, Guerrero Bustamante C, Boyle MM, Petrova ZO, Dedrick RM, Pope WH, Modlin RL, Hendrix RW, Hatfull GF, Science Education Alliance Phage Hunters Advancing Genomics and Evolutionary Science Sea-Phages Program. 2012. On the nature of mycobacteriophage diversity and host preference. Virology 434:187–201. doi:10.1016/j.virol.2012.09.026. 23084079 PMC3518647

[15] Dedrick R, Guerrero Bustamante C, Garlena RA, Russell DA, Ford K, Harris K, Gilmour KC, Soothill J, Jacobs-Sera D, Schooley RT, Hatfull GF, Spencer H. 2019. Engineered bacteriophages for treatment of a patient with a disseminated drug-resistant *Mycobacterium abscessus*. Nat Med 25:730–733. doi:10.1038/s41591-019-0437-z. 31068712 PMC6557439

[16] Marinelli LJ, Piuri M, Swigonova Z, Balachandran A, Oldfield LM, van Kessel JC, Hatfull GF. 2008. BRED: a simple and powerful tool for constructing mutant and recombinant bacteriophage genomes. PLoS One 3:e3957. doi:10.1371/journal.pone.0003957. 19088849 PMC2597740

[17] Dedrick RM, Smith BE, Garlena RA, Russell DA, Aull HG, Mahalingam V, Divens AM, Guerrero-Bustamante CA, Zack KM, Abad L, Gauthier CH, Jacobs-Sera D, Hatfull GF. 2021. *Mycobacterium abscessus* strain morphotype determines phage susceptibility, the repertoire of therapeutically useful phages, and phage resistance. mBio 12:e03431-20. doi:10.1128/mBio.03431-20. 33785625 PMC8092298

[18] Sassi M, Gouret P, Chabrol O, Pontarotti P, Drancourt M. 2014. Mycobacteriophage-drived diversification of Mycobacterium abscessus. Biol Direct 9:19. doi:10.1186/1745-6150-9-19. 25224692 PMC4172396

[19] Glickman C, Kammlade SM, Hasan NA, Epperson LE, Davidson RM, Strong M. 2020. Characterization of integrated prophages within diverse species of clinical nontuberculous mycobacteria. Virol J 17:124. doi:10.1186/s12985-020-01394-y. 32807206 PMC7433156

[20] Arndt D, Grant JR, Marcu A, Sajed T, Pon A, Liang Y, Wishart DS. 2016. PHASTER: a better, faster version of the PHAST phage search tool. Nucleic Acids Res 44:W16–21. doi:10.1093/nar/gkw387. 27141966 PMC4987931

[21] Ripoll F, Pasek S, Schenowitz C, Dossat C, Barbe V, Rottman M, Macheras E, Heym B, Herrmann JL, Daffe M, Brosch R, Risler JL, Gaillard JL. 2009. Non mycobacterial virulence genes in the genome of the emerging pathogen Mycobacterium abscessus. PLoS One 4:e5660. doi:10.1371/journal.pone.0005660. 19543527 PMC2694998

[22] Choi GE, Cho YJ, Koh WJ, Chun J, Cho SN, Shin SJ. 2012. Draft genome sequence of Mycobacterium abscessus subsp. bolletii BD(T). J Bacteriol 194:2756–2757. doi:10.1128/JB.00354-12. 22535937 PMC3347169

[23] Raiol T, Ribeiro GM, Maranhão AQ, Bocca AL, Silva-Pereira I, Junqueira-Kipnis AP, Brigido M.dM, Kipnis A. 2012. Complete genome sequence of Mycobacterium massiliense. J Bacteriol 194:5455. doi:10.1128/JB.01219-12. 22965084 PMC3457197

[24] Pope WH, Anders KR, Baird M, Bowman CA, Boyle MM, Broussard GW, Chow T, Clase KL, Cooper S, Cornely KA, Dejong RJ, Delesalle VA, Deng L, Dunbar D, Edgington NP, Ferreira CM, Weston Hafer K, Hartzog GA, Hatherill JR, Hughes LE, Ipapo K, Krukonis GP, Meier CG, Monti DL, Olm MR, Page ST, Peebles CL, Rinehart CA, Rubin MR, Russell DA, Sanders ER, Schoer M, Shaffer CD, Wherley J, Vazquez E, Yuan H, Zhang D, Cresawn SG, Jacobs-Sera D, Hendrix RW, Hatfull GF. 2014. Cluster M mycobacteriophages Bongo, PegLeg, and Rey with unusually large repertoires of tRNA isotypes. J Virol 88:2461–2480. doi:10.1128/JVI.03363-13. 24335314 PMC3958112

[25] Mavrich TN, Hatfull GF. 2019. Evolution of superinfection immunity in cluster A mycobacteriophages. mBio 10:e00971-19. doi:10.1128/mBio.00971-19. 31164468 PMC6550527

[26] Davidson RM, Hasan NA, Reynolds PR, Totten S, Garcia B, Levin A, Ramamoorthy P, Heifets L, Daley CL, Strong M. 2014. Genome sequencing of Mycobacterium abscessus isolates from patients in the United States and comparisons to globally diverse clinical strains. J Clin Microbiol 52:3573–3582. doi:10.1128/JCM.01144-14. 25056330 PMC4187745

[27] Jamet A, Touchon M, Ribeiro-Goncalves B, Carrico JA, Charbit A, Nassif X, Ramirez M, Rocha EPC. 2017. A widespread family of polymorphic toxins encoded by temperate phages. BMC Biol 15:75. doi:10.1186/s12915-017-0415-1. 28851366 PMC5576092

[28] Sun J, Siroy A, Lokareddy RK, Speer A, Doornbos KS, Cingolani G, Niederweis M. 2015. The tuberculosis necrotizing toxin kills macrophages by hydrolyzing NAD. Nat Struct Mol Biol 22:672–678. doi:10.1038/nsmb.3064. 26237511 PMC4560639

[29] Wang L, Jiang S, Deng Z, Dedon PC, Chen S. 2019. DNA phosphorothioate modification-a new multi-functional epigenetic system in bacteria. FEMS Microbiol Rev 43:109–122. doi:10.1093/femsre/fuy036. 30289455 PMC6435447

[30] Howard ST, Byrd TF, Lyons CR. 2002. A polymorphic region in Mycobacterium abscessus contains a novel insertion sequence element. Microbiology (Reading) 148:2987–2996. doi:10.1099/00221287-148-10-2987. 12368432

[31] Kim BJ, Kim BR, Hong SH, Seok SH, Kook YH, Kim BJ. 2013. Complete genome sequence of Mycobacterium massiliense clinical strain Asan 50594, belonging to the type II genotype. Genome Announc 1:e00429-13. doi:10.1128/genomeA.00429-13. 23833135 PMC3703596

[32] Matsumoto Y, Kinjo T, Motooka D, Nabeya D, Jung N, Uechi K, Horii T, Iida T, Fujita J, Nakamura S. 2019. Comprehensive subspecies identification of 175 nontuberculous mycobacteria species based on 7547 genomic profiles. Emerg Microbes Infect 8:1043–1053. doi:10.1080/22221751.2019.1637702. 31287781 PMC6691804

[33] Wetzel KS, Aull HG, Zack KM, Garlena RA, Hatfull GF. 2020. Protein-mediated and RNA-based origins of replication of extrachromosomal mycobacterial prophages. mBio 11:e00385-20. doi:10.1128/mBio.00385-20. 32209683 PMC7157519

[34] Hatfull GF. 2012. The secret lives of mycobacteriophages. Adv Virus Res 82:179–288. doi:10.1016/B978-0-12-394621-8.00015-7. 22420855

[35] Campbell A. 2003. Prophage insertion sites. Res Microbiol 154:277–282. doi:10.1016/S0923-2508(03)00071-8. 12798232

[36] Kim AI, Ghosh P, Aaron MA, Bibb LA, Jain S, Hatfull GF. 2003. Mycobacteriophage Bxb1 integrates into the Mycobacterium smegmatis groEL1 gene. Mol Microbiol 50:463–473. doi:10.1046/j.1365-2958.2003.03723.x. 14617171

[37] Smith MCM. 2015. Phage-encoded serine integrases and other large serine recombinases. Microbiol Spectr 3. doi:10.1128/microbiolspec.MDNA3-0059-2014. 26350324

[38] Ojha A, Anand M, Bhatt A, Kremer L, Jacobs WR, Jr., Hatfull GF. 2005. GroEL1: a dedicated chaperone involved in mycolic acid biosynthesis during biofilm formation in mycobacteria. Cell 123:861–873. doi:10.1016/j.cell.2005.09.012. 16325580

[39] Vuksanovic N, Zhu X, Serrano DA, Siitonen V, Metsa-Ketela M, Melancon CE, 3rd, Silvaggi NR. 2020. Structural characterization of three noncanonical NTF2-like superfamily proteins: implications for polyketide biosynthesis. Acta Crystallogr F Struct Biol Commun 76:372–383. doi:10.1107/S2053230X20009814. 32744249 PMC7397469

[40] Broussard GW, Oldfield LM, Villanueva VM, Lunt BL, Shine EE, Hatfull GF. 2013. Integration-dependent bacteriophage immunity provides insights into the evolution of genetic switches. Mol Cell 49:237–248. doi:10.1016/j.molcel.2012.11.012. 23246436 PMC3557535

[41] Zhang D, de Souza RF, Anantharaman V, Iyer LM, Aravind L. 2012. Polymorphic toxin systems: comprehensive characterization of trafficking modes, processing, mechanisms of action, immunity and ecology using comparative genomics. Biol Direct 7:18. doi:10.1186/1745-6150-7-18. 22731697 PMC3482391

[42] Mok BY, de Moraes MH, Zeng J, Bosch DE, Kotrys AV, Raguram A, Hsu F, Radey MC, Peterson SB, Mootha VK, Mougous JD, Liu DR. 2020. A bacterial cytidine deaminase toxin enables CRISPR-free mitochondrial base editing. Nature 583:631–637. doi:10.1038/s41586-020-2477-4. 32641830 PMC7381381

[43] Kim YS, Yang CS, Nguyen LT, Kim JK, Jin HS, Choe JH, Kim SY, Lee HM, Jung M, Kim JM, Kim MH, Jo EK, Jang JC. 2017. Mycobacterium abscessus ESX-3 plays an important role in host inflammatory and pathological responses during infection. Microbes Infect 19:5–17. doi:10.1016/j.micinf.2016.09.001. 27637463

[44] Laencina L, Dubois V, Le Moigne V, Viljoen A, Majlessi L, Pritchard J, Bernut A, Piel L, Roux AL, Gaillard JL, Lombard B, Loew D, Rubin EJ, Brosch R, Kremer L, Herrmann JL, Girard-Misguich F. 2018. Identification of genes required for Mycobacterium abscessus growth in vivo with a prominent role of the ESX-4 locus. Proc Natl Acad Sci U S A 115:E1002–E1011. doi:10.1073/pnas.1713195115. 29343644 PMC5798338

[45] Jamet A, Nassif X. 2015. New players in the toxin field: polymorphic toxin systems in bacteria. mBio 6:e00285-15. doi:10.1128/mBio.00285-15. 25944858 PMC4436062

[46] Gray TA, Derbyshire KM. 2018. Blending genomes: distributive conjugal transfer in mycobacteria, a sexier form of HGT. Mol Microbiol 108:601–613. doi:10.1111/mmi.13971. 29669186 PMC5997560

[47] Garvey P, Fitzgerald GF, Hill C. 1995. Cloning and DNA sequence analysis of two abortive infection phage resistance determinants from the lactococcal plasmid pNP40. Appl Environ Microbiol 61:4321–4328. doi:10.1128/AEM.61.12.4321-4328.1995. 8534099 PMC167743

[48] Dumas E, Christina Boritsch E, Vandenbogaert M, Rodriguez de la Vega RC, Thiberge JM, Caro V, Gaillard JL, Heym B, Girard-Misguich F, Brosch R, Sapriel G. 2016. Mycobacterial pan-genome analysis suggests important role of plasmids in the radiation of type VII secretion systems. Genome Biol Evol 8:387–402. doi:10.1093/gbe/evw001. 26748339 PMC4779608

[49] Ummels R, Abdallah AM, Kuiper V, Aajoud A, Sparrius M, Naeem R, Spaink HP, van Soolingen D, Pain A, Bitter W. 2014. Identification of a novel conjugative plasmid in mycobacteria that requires both type IV and type VII secretion. mBio 5:e01744-14. doi:10.1128/mBio.01744-14. 25249284 PMC4173767

[50] Davis JJ, Wattam AR, Aziz RK, Brettin T, Butler R, Butler RM, Chlenski P, Conrad N, Dickerman A, Dietrich EM, Gabbard JL, Gerdes S, Guard A, Kenyon RW, Machi D, Mao C, Murphy-Olson D, Nguyen M, Nordberg EK, Olsen GJ, Olson RD, Overbeek JC, Overbeek R, Parrello B, Pusch GD, Shukla M, Thomas C, VanOeffelen M, Vonstein V, Warren AS, Xia F, Xie D, Yoo H, Stevens R. 2020. The PATRIC Bioinformatics Resource Center: expanding data and analysis capabilities. Nucleic Acids Res 48:D606–D612. doi:10.1093/nar/gkz943. 31667520 PMC7145515

[51] Petrova ZO, Broussard GW, Hatfull GF. 2015. Mycobacteriophage-repressor-mediated immunity as a selectable genetic marker: adephagia and BPs repressor selection. Microbiology (Reading) 161:1539–1551. doi:10.1099/mic.0.000120. 26066798 PMC4681040

[52] Amarh ED, Dedrick RM, Garlena RA, Russell DA, Jacobs-Sera D, Hatfull GF. 2021. Genome sequence of Mycobacterium abscessus phage phiT46-1. Microbiol Resour Announc 10:e01421-20. doi:10.1128/MRA.01421-20. 33446600 PMC7849713

[53] Lee MH, Pascopella L, Jacobs WR, Jr., Hatfull GF. 1991. Site-specific integration of mycobacteriophage L5: integration-proficient vectors for Mycobacterium smegmatis, Mycobacterium tuberculosis, and bacille Calmette-Guerin. Proc Natl Acad Sci U S A 88:3111–3115. doi:10.1073/pnas.88.8.3111. 1901654 PMC51395

[54] Jiang H, Lei R, Ding SW, Zhu S. 2014. Skewer: a fast and accurate adapter trimmer for next-generation sequencing paired-end reads. BMC Bioinformatics 15:182. doi:10.1186/1471-2105-15-182. 24925680 PMC4074385

[55] Wick RR, Judd LM, Gorrie CL, Holt KE. 2017. Unicycler: resolving bacterial genome assemblies from short and long sequencing reads. PLoS Comput Biol 13:e1005595. doi:10.1371/journal.pcbi.1005595. 28594827 PMC5481147

[56] Gordon D, Abajian C, Green P. 1998. Consed: a graphical tool for sequence finishing. Genome Res 8:195–202. doi:10.1101/gr.8.3.195. 9521923

[57] Gordon D, Green P. 2013. Consed: a graphical editor for next-generation sequencing. Bioinformatics 29:2936–2937. doi:10.1093/bioinformatics/btt515. 23995391 PMC3810858

[58] Sovic I, Sikic M, Wilm A, Fenlon SN, Chen S, Nagarajan N. 2016. Fast and sensitive mapping of nanopore sequencing reads with GraphMap. Nat Commun 7:11307. doi:10.1038/ncomms11307. 27079541 PMC4835549

[59] Kaas RS, Leekitcharoenphon P, Aarestrup FM, Lund O. 2014. Solving the problem of comparing whole bacterial genomes across different sequencing platforms. PLoS One 9:e104984. doi:10.1371/journal.pone.0104984. 25110940 PMC4128722

[60] Huson DH. 1998. SplitsTree: analyzing and visualizing evolutionary data. Bioinformatics 14:68–73. doi:10.1093/bioinformatics/14.1.68. 9520503

[61] Cresawn SG, Bogel M, Day N, Jacobs-Sera D, Hendrix RW, Hatfull GF. 2011. Phamerator: a bioinformatic tool for comparative bacteriophage genomics. BMC Bioinformatics 12:395. doi:10.1186/1471-2105-12-395. 21991981 PMC3233612

[62] Dedrick RM, Mavrich TN, Ng WL, Cervantes Reyes JC, Olm MR, Rush RE, Jacobs-Sera D, Russell DA, Hatfull GF. 2016. Function, expression, specificity, diversity and incompatibility of actinobacteriophage parABS systems. Mol Microbiol 101:625–644. doi:10.1111/mmi.13414. 27146086 PMC4998052

[63] Pope WH, Mavrich TN, Garlena RA, Guerrero-Bustamante CA, Jacobs-Sera D, Montgomery MT, Russell DA, Warner MH, Hatfull GF, Science Education Alliance-Phage Hunters Advancing Genomics and Evolutionary Science (SEA-PHAGES). 2017. Bacteriophages of Gordonia spp. display a spectrum of diversity and genetic relationships. mBio 8:e01069-17. doi:10.1128/mBio.01069-17. 28811342 PMC5559632

