# Supplementary material for: The Prophage and Plasmid Mobilome as a Likely Driver of Mycobacterium abscessus Diversity
Source: mBio. 2021 Mar 30;12(2):e03441-20. doi: 10.1128/mBio.03441-20 (PMC8092301; doi:10.1128/mBio.03441-20)
Supplement: TABLE S1 [file mBio.03441-20-st001.pdf]

Table S1. *attB* sites in *M. abscessus*

	Core sequence[1]	Int	attB	attB	ATCC1997 coordinates
MabA-1	GGGGCGGTAGCTCAGTTGGTTAGAGCCGTGGACTCATAATCC (e.g. GD11-1)	Int-Y	attB-5	Mab_t5028; 5' half of tRNA-met	1754373 (tRNA-met)
	CTGGTGCGCCGTCAGGGGCTCGAACCCCGGACCCGCTGATTAAGAGTC (GD43A-1)	Int-Y	attB-18	Mab_t5022c; 3' end tRNA-Lys	1550157
MabA-2	GCGGACTTAAAATCCGCCAAGTGTCGGTTCGAGTCCGACTGGGGGCAC	Int-Y	attB-15	Mab_t5019; tRNA-Leu	1191297
MabA-3	GGGGCGGTAGCTCAGTTGGTTAGAGCCGTGGACTCATAATCC	Int-Y	attB-5	Mab_t5028; 5' half of tRNA-met	1754373 (tRNA-met)
MabB	GTAATGAATAGGTCAGGGGTTCGATTCCCCTGGGTGGCTC	Int-Y	attB-2	Mab_t5010; 3' end tRNA-Thr	490925 (tRNA-thr)
MabC	TACTCGTGAGTAAGAACT (e.g. GD13-2)	Int-Y	attB-13	Extreme 5' end of Mab_3947	3995698
	TACTTTCGA**G**TAAGGTA (e.g. GD33-1)		attB-12	Extreme 5' end of Mab_3824	3869544
MabD	CGAGAAGGTCAGGGGTTCGATTCCCCTTAGCTCCACCAA (GD17)	Int-Y	attB-3	Mab_t5017; 3' end tRNA-ala	1102715
	C**A**GAGACAAAAAATCCCAGG**T**CGTCTGACCT**G**GGATTATGGTGGAGCTAAGGGGACTCGAA<br>CCCCTGACCCCCACA (GD12-2, GD05-1)		attB-10	Mab_t5041; 3' tRNA-ala	3491796
MabE-1	AGCATCCGACTGTTAATCGGACGGTTATTGGTTCGAGTCCAATCGGGGGAGC (GD04-1)	Int-Y	attB-4	Mab_t5027 (tRNA-asn)	1739368
MabE-2	CCCAGAGGTCGCAGGTTCAAATCCTGTCCCCGCTACTAGGT	Int-Y	attB-16	Mab_t5042; tRNA-met	3537993
MabF	ATCGAGAAGGTCAGGGGTTCGATTCCCCTTAGCTCCAC (GD08-3)		attB-3	Mab_t5017; 3' end tRNA-ala	1102713 (tRNA-ala)
MabG	CGGGTTCAATTCCCGGCAGCTCCAC	Int-Y	attB-11	Intergenic: 3471-3472 (550 bp)	3513406 (ATCC)
MabH	ATT	Int-Y	attB-8	Extreme 3' end of Mab_2979	3039287
MabI	GACTA (GD54-2)	Int-S	attB-9	In Mab_3230c	3265145
	GGGCT (GD86-1)		attB-17	In Mab_3265c	3302860
MabJ	AAGTCGTA	Int-S	attB-7	In 5' end of Mab_2445	2502029
MabK[2]	CAGAAGGTTAGGGGTTCGAATCCCTTCGGGCGCACCAT	Int-Y	attB-1	Mab_t5006; 3' end tRNA-arg	233517 (tRNA-arg)
MabL	AGGGGTTCGAGTCCCCTTAGCTCCACCATA (GD88-1)	Int-Y	attB-10	Mab_t5041c; 3' end tRNA-ala	3491832 (tRNA-ala)
	AGGGGTTCGAGTCCCCTTAGCTCCAC (Bolletii)				3491836 (tRNA-ala)
MabM	CGGGTTCAATTCCCGGCAGCTCCAC	Int-Y	attB-11	Intergenic Mab_3471-3472	3513406
MabN	TACTCGTGAGTAAGAACT	Int-Y	attB-13	Extreme 5' end of Mab_3947	3995689
MabO	TACTCTGGAGTAAG	Int-Y	attB-14	Inter Mab_4442c - 4443	4519114
MabP	CTGGGGGTCAAGTGGTCGCAGGTTCAAATCCTGTCAGCCCGAC	Int-Y	attB-6	MAB_t5033; tRNA-pro	2431284
MabQ	CGGACGGTT**A**TTGGTTCGAGTCCAA**T**CGGGGGAG	Int-Y	attB-4	Mab_t5027; 3' end tRNA-asn	1739385

[1]The common core sequence shared between *attP*, *attB*, *attL* and *attR* is shown; mismatches are in bold type.
[2]The attB-1 core is present once in most strains, but twice in ATCC19977 due to insertion of a 16.5 kbp segment, containing some phage-related sequences.
