# Supplementary material for: The Prophage and Plasmid Mobilome as a Likely Driver of Mycobacterium abscessus Diversity
Source: mBio. 2021 Mar 30;12(2):e03441-20. doi: 10.1128/mBio.03441-20 (PMC8092301; doi:10.1128/mBio.03441-20)
Supplement: TABLE S2 [file mBio.03441-20-st002.pdf]

Table S2. Predicted *M. abscessus* prophage immunity groups

**Immunity[1]**	**Cluster**	**Prophages[8]**	**attB**	**Int[6]**	**Int-dep Imm?[7]**
Imm-1	MabA1	prophiATCC19977-1, prophiGD15-1, prophiGD17-2, prophiGD102-1, *prophiGD12-1*, *prophiGD14-1*, *prophiGD23-1, prophiGD39-1*, *prophiGD84-1*	attB-5	Int-Y	No
Imm-2	MabA1	prophiGD20-1, prophiGD21-2, prophiGD22-1, prophiGD43A-1, prophiGD57-2, *prophiGD08-1*, *prophiGD13-1*, *prophiGD24-1*, *prophiGD35-1*, *prophiGD36-1*, *prophiGD42-1*, *prophiGD75-2*, *prophiGD100A-1, prophiGD100B-1*	attB-5	Int-Y	No
Imm-3	MabA1	prophiGD27-1	attB-5	Int-Y	No
Imm-4	MabA2,	prophiGD02-2, prophiGD90-1, *prophiGD16-2*	attB-15	Int-Y	No
	MabA3	prophiGD91-2	attB-5	Int-Y	No
Imm-5	MabA1	*prophiGD10-1, prophiGD30-1, prophiGD58-2*	attB-5	Int-Y	No
Imm-6	MabA1	prophiGD11-1	attB-5	Int-Y	No
Imm-7	MabB	prophiGD08-2, prophiGD11-2, prophiGD21-1, prophiGD42-2, prophiGD89-1	attB-2	Int-Y	Yes
Imm-8	MabB	prophiGD34-2, prophiGD43A-2, prophiGD62-1	attB-2	Int-Y	Yes
Imm-9[2]	MabB	prophiGD16-1	attB-2	Int-Y	Yes
Imm-10	MabC	prophiGD13-2, prophiGD39-2, prophiGD44-1, prophiGD51-1, prophiGD52-1, prophiGD57-1, prophiGD91-1, prophiGD104-2, *prophiGD56-1*, *prophiGD61-1*, *prophiGD72-1*	attB-13	Int-Y	Yes
Imm-11	MabC	prophiGD33-1, prophiGD43A-3, prophiGD100A-2, *prophiGD42-3*	attB-12	Int-Y	Yes
Imm-12	MabD	prophiGD05-1, prophiGD17-1	attB-10	Int-Y	No
Imm-13	MabD	prophGD12-2, *prophiGD84-2*	attB-10	Int-Y	No
Imm-14	MabE1	prophiGD25-1, prophiGD54-1, prophiGD102-2, *prophiGD09-1, prophiGD45-1, prophiGD52-2*	attB-4	Int-Y	No
Imm-15	MabE1	*prophiGD53-1*, prophiGD68-1, prophiGD04-1, prophiGD111-1	attB-4	Int-Y	No
Imm-16	MabE2	prophiGD91-4	attB-6	Int-Y	No
Imm-17	MabF	prophiGD08-3, prophiGD11-3, prophiGD62-2	attB-3	Int-Y	No
Imm-18	MabG	prophiGD03-1	attB-11	Int-Y	No
Imm-19	MabG	prophiGD21-3, prophiGD24-2, prophiGD95-1	attB-11	Int-Y	No
Imm-20	MabG	prophiGD58-1	attB-11	Int-Y	No
Imm-21	MabH	prophiGD05-2, prophiGD36-2	attB-8	Int-Y	Yes
Imm-22	MabI	prophiGD54-2	attB-9	Int-S	No
		prophiGD86-1	attB-17	Int-S	No
Imm-23	MabJ	prophiGD24-3, prophiGD43A-4, *prophiGD21-4, prophiGD75-1*	attB-7	Int-S	No
Imm-24	MabK	prophiGD43A-5	attB-1	Int-Y	Yes
Imm-25	MabL	prophiBoletti-1	attB10	Int-Y	No
Imm-26	MabL	prophiGD43A-6, prophiGD88-1, *prophiGD55-1, prophiGD60-1*	attB-10	Int-Y	No
Imm-27[3]	MabM	prophiGD05-3	attB-11	Int-Y	No
Imm-28[4]	MabN	prophiGD53-3, prophiGD62-3, prophiGD69-1, prophiGD108-1, *prophiGD02-1 prophiGD95-2*	attB-13	Int-Y	Yes
Imm-28[5]	MabO	prophiGD91-3	attB-14	Int-Y	Yes
Imm-29	MabP	prophiGD51-2	attB-6	Int-Y	No
Imm-30	MabQ	prophiGD79-1	attB-4	Int-Y	No

[1]Immunity groups were defined as prophages sharing similar repressors, typically having >90% amino acid identity. Prophages in different immunity groups typically share <70% amino acid identity.

[2]The MabB (Imm-9) repressor in prophiGD16-1 is overall 88% aa identical to Imm-7 phages, but differs substantially in the HTH region and is likely heteroimmune.

[3]The MabM repressor is 79% amino acid identity to Cluster MabG genomes, and heteroimmunity is uncertain.

[4]The prophGD53-3 repressor is 89% identical to other MabN's, and heteroimmunity is plausible.

[5]MabO repressor has 81% aa identity to imm-26 repressors, but it very similar in the putative HTH region, and homoimmunity is likely.

[6]Integrase is designated as being in the tyrosine-integrase (Int-Y) family or serine-integrase family (Int-S)

[7]Integration-dependent immunity systems are those in which the *attP* core sequence is within the repressor gene such that the gene is interrupted in the prophage state.

[8]Repressors identified in incomplete prophages are shown in italic type
