# Supplementary material for: The Prophage and Plasmid Mobilome as a Likely Driver of Mycobacterium abscessus Diversity
Source: mBio. 2021 Mar 30;12(2):e03441-20. doi: 10.1128/mBio.03441-20 (PMC8092301; doi:10.1128/mBio.03441-20)
Supplement: TABLE S3 [file mBio.03441-20-st003.pdf]

Table S3. Types of Polymorphic Toxin-Immunity configurations

Tox-Imm config.	Prototype (cluster)	Others
a	prophiGD57-1 (MabC)	prophiGD33-1 (MabC), prophiGD39-2 (MabC), prophiGD43A-3 (MabC), prophiGD100A-2 (MabC)
b	prophiGD08-3 (MabF)	prophiGD11-3 (MabF), prophiGD62-2 (MabF)
c	prophiGD21-3 (MabG)	prophiGD24-2 (MabG, prophiGD58-1 (MabG)
d	prophiGD43A-5 (MabK)	None
e	prophiGD43A-6 (MabL)	prophiGD88-1 (MabL)
f	prophiGD05-3 (MabM)	None
g	prophiGD03-1 (MabG)	None
h	prophiGD43A-1 (MabA1)	None
i	prophiGD04-1 (MabE1)	prophiGD68-1 (MabE1) , prophiGD25-1 (MabE1), prophiGD54-1 (MabE1), prophiGD102-2 (MabE1)
j	prophiGD91-4 (MabE2)	None
k	prophiGD54-2 (MabI)	prophiGD54-2 (MabI)
l	prophiGD12-2 (MabD)	prophiGD05-1 (MabD)
m	prophiGD108-1 (MabN)	prophiGD62-3 (MabN)
n	prophiGD91-2 (MabA3)	None
o	prophiGD21-1 (MabB)	prophiGD08-2 (MabB), prophiGD11-2 (MabB), prophiGD42-2 (MabB), prophiGD05-2 (MabH)
p	prophiGD91-3 (MabO)	None
q	prophiGD34-2 (MabB)	prophiGD62-1 (MabB)
r	prophiGD36-2 (MabH)	prophiGD05-2 (MabH)
s	prophiGD89-1 (MabB)	prophiGD16-1 (MabB), prophiGD43A-2 (MabB)
t	prophiGD13-2 (MabC)	prophiGD44-1 (MabC), prophiGD51-1 (MabC), prophiGD52-1 (MabC), prophiGD91-1 (MabC), prophiGD104-2 (MabC)
u	prophiGD17-1 (MabD)	None
