# Supplementary material for: The Prophage and Plasmid Mobilome as a Likely Driver of Mycobacterium abscessus Diversity
Source: mBio. 2021 Mar 30;12(2):e03441-20. doi: 10.1128/mBio.03441-20 (PMC8092301; doi:10.1128/mBio.03441-20)
Supplement: FIG S1 [file mBio.03441-20-sf001.pdf]

MabB

phiGD21-1
R P G L P L P D G A T Q G N R T P D L F I T S L T K R A K T S P S P F V P S V V P P S D P D R I A A *
201 AGGCCGGGCCTGCCACTCCCCGATGGAGCCACCCAGGGGAATCGAACCCCTGACCTATTCATTACAAGTCTGACAAAGAGGGCCAAAACGTCACCCTCTCCATTTGTCCCGAGTGTGGTGCCGCCCTCTGACCCCGATCGGATCGCGGCGTGA

prophiGD21-1
R P G L P L P D G A T Q G N R T P D L F I T S E L L Y R L S *
701 AGGCCGGGCCTGCCACTCCCCGATGGAGCCACCCAGGGGAATCGAACCCCTGACCTATTCATTACGAGTGAATTGCTCTACCGACTGAGCTAGGGTGGCGCGCCGGGCGAACCCGGCGCCCGAGAGTCTACGGCAGACACCCTGGCAGACC
.:....|....:....|....:....|....:....|....:....|....:....|....:....|....:....|....:....|....:....|....:....|....:....|....:....|....:....|....:....|....
TCCGGCCCGGACGGTGAGGGGCTACCTCGGTGGGTCCCCTTAGCTTGGGGACTGGATAAGTAATGCTCACTTAACGAGATGGCTGACTCGATCCCACCGCGCGGCCCGCTTGGGCCGCGGGCTCTCAGATGCCGTCTGTGGGACCGTCTGG

phiGD34-2
D S Q P D P E P P R G I E P L T Y S L L V G R T R S F R H T I T S A R N V V L P S D P D R I A A *
201 GACTCCCAGCCAGACCCAGAGCCACCCAGGGGAATCGAACCCCTGACCTATTCATTACTAGTGGGACGAACCCGGAGCTTTCGTCACACTATTACTTCTGCCCGAAATGTGGTGCTGCCCTCTGACCCCGATCGGATCGCGGCCTGA

prophiGD34-2
D S Q P D P E P P R G I E P L T Y S L R V N C S T D *
401 GACTCCCAGCCAGACCCAGAGCCACCCAGGGGAATCGAACCCCTGACCTATTCATTACGAGTGAATTGCTCTACCGACTGAGCTAGGGTGGCGCGCCGGGCGAACCCGGCGCCCGAGAGTCTACGGCAGACACCCT
....:....|....:....|....:....|....:....|....:....|....:....|....:....|....:....|....:....|....:....|....:....|....:....|....:....|....:.
CTGAGGGTCGGTCTGGGTCTCGGTGGGTCCCCTTAGCTTGGGGACTGGATAAGTAATGCTCACTTAACGAGATGGCTGACTCGATCCCACCGCGCGGCCCGCTTGGGCCGCGGGCTCTCAGATGCCGTCTGTGGGA

phiGD16-1
R P G L P L P D G A T Q G N R T P D L F I T S L T K R A K T S P S P F V P S V V P P S D P D R I A A *
301 CGGCCGGGTCTGCCTCTACCCGATGGAGCCACCCAGGGGAATCGAACCCCTGACCTATTCATTACAAGTCTGACAAAGAGGGCCAAAACGTCACCCTCTCCATTTGTCCCGAGTGTAGTGCCGCCCTCTGACCCCGATCGGATCGCGGCGTAA

prophiGD16-1
R P G L P L P D G A T Q G N R T P D L F I T S E L L Y R L S *
501 CGGCCGGGTCTGCCTCTACCCGATGGAGCCACCCAGGGGAATCGAACCCCTGACCTATTCATTACGAGTGAATTGCTCTACCGACTGAGCTAGGGTGGCGCGCCGGGCGAACCCGGCGCCCGAGAGTCTACGGCAGACACCCTGGCAGACCCA
....:....|....:....|....:....|....:....|....:....|....:....|....:....|....:....|....:....|....:....|....:....|....:....|....:....|....:....|...
GCCGGCCCAGACGGAGATGGGCTACCTCGGTGGGTCCCCTTAGCTTGGGGACTGGATAAGTAATGCTCACTTAACGAGATGGCTGACTCGATCCCACCGCGCGGCCCGCTTGGGCCGCGGGCTCTCAGATGCCGTCTGTGGGACCGTCTGGGT

MabC

phiGD57-1
G G Q L A T E G A S S T V L T H E Y E P Y K S A F R R A S S G H G F R S G K Q L T C N K S T R S V S *
201 GGCGGGCAGCTAGCCACGGAGGGGGCGAGTAGCACAGTTCTTACTCACGAGTATGAACCGTATAAGTCCGCGTTCCGGCGCGCCAGCTCAGGTCACGGTTTCCGCTCGGGGAAGCAACTGACCTGCAACAAGTCCACCCGTTCGGTTTCCTAG

prophiGD57-1
G G Q L A T E G A S S T V L T H E *
501 GGCGGGCAGCTAGCCACGGAGGGGGCGAGTAGCACAGTTCTTACTCACGAGTAAGAACTGTATGCCTCTTTCGATGCGTGCGAAATGTTGGGTCCCGGTGACGTATG

'phiGD33-1'
S D T Y G D A Y L I R K Y G S R I C K P A D G G R L G V A S H R F A S A G F L S C N N S R H P V S K *
201 TCGGACACCTACGGGGACGCTTACCTTA**T**TCGAAAGTATGGTTCGCGAATTTGTAAACCCGCTGATGGAGGGCGGTTGGGGGTCGCTAGTCACCGGTTCGCATCGGCGGGGTTTCTGTCCTGCAACAACTCCCGGCATCCGGTTTCTAAGTGA

prophiGD33-1
S D T Y G D A Y L I R K *
701 TCGGACACCTACGGGGACGCTTACCTTATTCGAAAGTAAGATCAGCGCGAATACTGCTGCGAACTCGCCCGGGCTACGG

MabK

```
'phiGD43A-5'
    D  G  P  M  V  R  P  K  G  F  E  P  L  T  F  W  S  V  V  R  N  P  L  T  C  I  D  T  E  P  T  D  L  V  A  H  R  S  R  R  R  A  A  E  L  E  A  D  A  A  *
201 GACGGTCCTATGGTGCGCCCGAAGGGATTCGAACCCCTAACCTTCTGGTCAGTAGTTCGAAACCCGTTGACCTGCATCGACACCGAGCCTACAGACCTAGTAGCGCACCGCAGCAGGCGGCGCGCAGCAGAACTCGAAGCCGATGCCGCATGA

prophiGD43A-5
     D  G  P  M  V  R  P  K  G  F  E  P  L  T  F  *
801  GACGGTCCTATGGTGCGCCCGAAGGGATTCGAACCCCTAACCTTCTGATCCGTAGTCAGATGCTCTATCCGTTGAGCTACGGGCGCTTACTATTCAATTGTTGCAGGTCAACAGGTTTGGCCTGCAGACCGA
     ....:....|....:....|....:....|....:....|....:....|....:....|....:....|....:....|....:....|....:....|....:....|
     CTGCCAGGATACCACGCGGGCTTCCCTAAGCTTGGGGATTGGAAGACTAGGCATCAGTCTACGAGATAGGCAACTCGATGCCCGCGAATGATAAGTTAACAACGTCCAGTTGTCCAAACCGGACGTCTGGCT
```

MabN

```
'phiGD53-3'
    K  G  D  Q  L  T  R  G  V  G  N  N  T  V  L  T  H  E  Y  E  P  Y  K  R  I  G  R  R  A  S  S  G  H  I  F  R  S  G  K  Y  L  T  C  N  N  D  D  I  K  I  T  *
201 AAGGGTGATCAGCTAACTCGGGGAGTGGGGAACAATACAGTTCTTACTCACGAGTATGAACCGTATAAGCGCATCGGTCGTCGCGCGAGTTCGGGTCACATTTTCCGCTCGGGAAAGTATCTGACCTGCAACAACGACGACATCAAGATCACGT

prophiGD53-3
    K  G  D  Q  L  T  R  G  V  G  N  N  T  V  L  T  H  E  *
401 AAGGGTGATCAGCTAACTCGGGGAGTGGGGAACAATACAGTTCTTACTCACGAGTAAGAACTGTATGCCTCTTTCGATGCGCGCGAAATGTTGGGTCCTGGTGACGTATGTCTCGCCCGGTTTCGGCCACCGGTGGTGTCCTACGCTGGCCTCA
```

MabO

```
'phiGD91-3'
    S  E  G  G  R  N  A  I  L  T  P  E  Y  E  S  R  K  S  W  V  S  R  S  V  Q  G  H  G  L  R  S  G  K  Y  L  T  C  N  N  R  R  I  K  I  T  *
501 TCTGAGGGCGGCAGGAACGCTATTCTTACTCCAGAGTATGAATCGCGTAAGTCGTGGGTGAGCCGGAGTGTTCAGGGTCACGGTTTGCGATCAGGGAAGTATCTGACCTGCAACAACCGTCGAATCAAGATCACGTAG

prophiGD91-3
    S  E  G  G  R  N  A  I  L  T  P  E  *
701 TCTGAGGGCGGCAGGAACGCTATTCTTACTCCAGAGTAAGAATGCTCGGTAATCCTGCCCCCTCAACAGTGGAGAGACAAGTGGCAACTAGCTCGGCACGCAAGTCAGACGCTTCGCAAGGC
```

MabH

```
prophiGD36-2
                            I  N  S  I  C  L  T  F  Q  P  S  P  *
 CCCCACGCCGACCGATCAGCGTTACTGCATCAACTCAATTTGTCTGACGTTGCAGCCGAGCCCGTAGGGTC   - attL
TCGACCAGCGCCCTGACCTCGCTTACTGAAGCAACTCGATTAGTCTGCGGTTGGCACCACAGTAACGTCCCAATCGGCGG - attR

                                     X

                            I  N  S  I  S  L  R  L  A  P  Q  *
 CCCCACGCCGACCGATCAGCGTTACTGCATCAACTCAATTAGTCTGCGGTTGGCACCACAGTAACGTCCCAATCGGCGG - attB
TCGACCAGCGCCCTGACCTCGCTTACTGAAGCAACTCGATTTGTCTGACGTTGCAGCCGAGCCCGTAGGGTC   - attP
```

Figure S1
